# Aligned macrocycle pores in ultrathin films for accurate molecular sieving

**DOI:** 10.1038/s41586-022-05032-1

**Published:** 2022-08-31

**Authors:** Zhiwei Jiang, Ruijiao Dong, Austin M. Evans, Niklas Biere, Mahmood A. Ebrahim, Siyao Li, Dario Anselmetti, William R. Dichtel, Andrew G. Livingston

**Affiliations:** 1 Barrer Centre, Department of Chemical Engineering, Imperial College London, London, UK; 2 Department of Engineering and Materials Science, Queen Mary University of London, London, UK; 3 Shanghai Centre for Systems Biomedicine, Key Laboratory of Systems Biomedicine (Ministry of Education), Shanghai Jiao Tong University, Shanghai, China; 4 Department of Chemistry, Northwestern University, Evanston, IL USA; 5 George & Josephine Butler Polymer Research Laboratory, Center for Macromolecular Science & Engineering, Department of Chemistry, University of Florida, Gainesville, FL, USA; 6 Experimental Biophysics and Applied Nanoscience, Faculty of Physics, Bielefeld University, Bielefeld, Germany

**Keywords:** Chemical engineering, Chemical engineering, Porous materials, Materials chemistry

## Abstract

Polymer membranes are widely used in separation processes including desalination^1^, organic solvent nanofiltration^2,3^ and crude oil fractionation^4,5^. Nevertheless, direct evidence of subnanometre pores and a feasible method of manipulating their size is still challenging because of the molecular fluctuations of poorly defined voids in polymers^6^. Macrocycles with intrinsic cavities could potentially tackle this challenge. However, unfunctionalized macrocycles with indistinguishable reactivities tend towards disordered packing in films hundreds of nanometres thick^7–9^, hindering cavity interconnection and formation of through-pores. Here, we synthesized selectively functionalized macrocycles with differentiated reactivities that preferentially aligned to create well-defined pores across an ultrathin nanofilm. The ordered structure was enhanced by reducing the nanofilm thickness down to several nanometres. This orientated architecture enabled direct visualization of subnanometre macrocycle pores in the nanofilm surfaces, with the size tailored to ångström precision by varying the macrocycle identity. Aligned macrocycle membranes provided twice the methanol permeance and higher selectivity compared to disordered counterparts. Used in high-value separations, exemplified here by enriching cannabidiol oil, they achieved one order of magnitude faster ethanol transport and threefold higher enrichment than commercial state-of-the-art membranes. This approach offers a feasible strategy for creating subnanometre channels in polymer membranes, and demonstrates their potential for accurate molecular separations.

## Abstract (web summary)

Selectively functionalized macrocycles were synthesized with reactivities that preferentially aligned to create well-defined pores across an ultrathin nanofilm offering a strategy to create subnanometre channels in polymer membranes, and demonstrating potential for accurate molecular separations.

## Main

The key feature of most separation membranes is their pore structure, and a much sought-after prize is precise control over pore size; yet until now we simply do not have a fundamental understanding of the geometry of subnanometre pores, or accurate control of their size^10,11^. In conventional polymeric membranes, subnanometre pores arise from interconnected microvoids produced by either the packing of linear polymers or the network structures of crosslinked polymers. Linear polymers of intrinsic microporosity provide high free volume microporosity owing to their rigid backbones^6^, but suffer from physical ageing and polymer relaxation, which lead to pore collapse^12^. Crosslinked polymer networks fabricated by interfacial polymerization have demonstrated enduring membrane performance^2^, but the rapid and stochastic crosslinking reaction makes it hard to accurately control microvoid architecture.

**Fig. 1:**
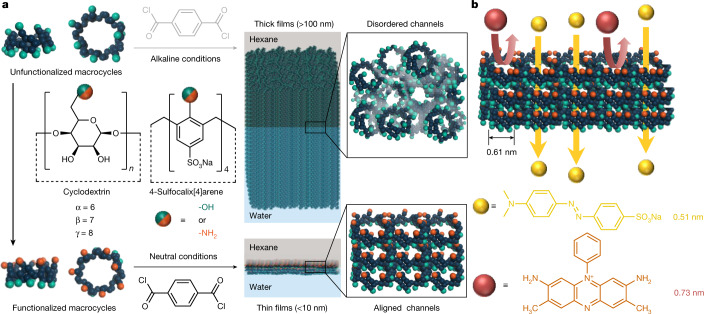
Fabrication of ultrathin polyamide nanofilms incorporating aligned macrocycles. **a**, Amino-functionalized macrocycles synthesized from unfunctionalized hydroxyl precursors, cyclodextrin or 4-sulfocalix[4]arene sodium salt. An ultrathin polyamide nanofilm incorporating aligned macrocycle channels was created at a free interface between an aqueous solution containing amino macrocycles and a hexane solution containing acyl chloride, in contrast to a thick film incorporating disordered channels fabricated from the unfunctionalized macrocycles. **b**, Schematic demonstrating molecular sieving through nanofilms incorporating aligned macrocycles.

Porous materials, including covalent organic frameworks (COFs)^13^, metal organic frameworks (MOFs)^14^ and porous organic cages (POCs)^15^, could potentially have their intrinsic cavities/apertures translated into membrane pores, but previous work has confronted the inevitable barriers of grain boundaries or disordered packing. Recently, macrocycles with permanent cavities, such as cyclodextrins, have been crosslinked into polyester separating layers through interfacial polymerization^7,8^. The cavities were assumed to be preserved as intrinsic membrane pores. However, because both the wide and narrow rims of unfunctionalized cyclodextrins were enriched with hydroxyl groups of similar reactivity under alkaline conditions, random crosslinking occurred during interfacial reaction and created more than 100-nm-thick films^7,8^ (Fig. 1a). Macrocycles with amines of indistinguishable reactivity also tend to react and pack stochastically during vigorous interfacial polymerization^9^. This non-selective crosslinking reduces the possibility that adjacent cavities in the macrocycles form aligned through-pores, and explains unexpectedly high rejections of molecules smaller than the cavity size^7,8^. Essentially, the uniform macrocycle cavity size was not translated into the uniform membrane pore size required to achieve sharp selectivity between different solutes.

## Ultrathin films with aligned macrocycles

The challenge is to arrange the porous materials in an ordered orientation, so that their interior cavities are aligned to provide straight subnanometre percolation channels. Here, we align amino-functionalized macrocycles in ultrathin nanofilms to create well-defined subnanometre pores for accurate molecular sieving in organic solvent nanofiltration (OSN). Amino-functionalized macrocycles were synthesized by selectively functionalizing the primary hydroxyls on the upper (narrow) rims of cyclodextrins and 4-sulfocalix[4]arene sodium salt (SC[4]A) to highly reactive amine groups, with the lower (wide) rims unchanged (Fig. 1a and Supplementary Fig. 1). Nuclear magnetic resonance (NMR) and Fourier-transform infrared (FTIR) spectroscopy confirmed their formation (Supplementary Figs. 2–22 and Table 1). As the long and flexible urethane linkage reduced intramolecular hydrogen bonding, these amino-functionalized macrocycles showed good solubility in water at neutral conditions^16^. When polymerized at a free interface with acyl chloride (for example, terephthaloyl chloride, TPC), the upper rim with highly reactive amines preferentially faces up into the organic phase where the crosslinking reaction occurs and the unreactive lower rim faces downwards into the aqueous solution (Supplementary Fig. 23). Macrocycles are then preferentially aligned through the crosslinked ultrathin nanofilm to form well-defined subnanometre channels, which can provide precise molecular sieving for solutes with a size difference as low as 0.2 nm (Fig. 1b). By contrast, other synthetic routes produce water-insoluble derivatives that require alkaline conditions^17^. This deprotonates the hydroxyl groups at the lower rim and triggers the crosslinking reaction, which packs macrocycles randomly.

**Fig. 2:**
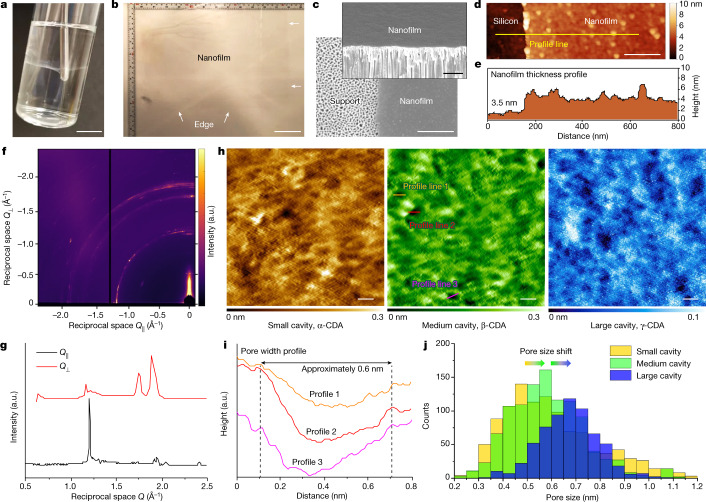
Characterization of ultrathin polyamide nanofilms incorporating aligned macrocycle pores. **a**, Photograph of a nanofilm formed at a free interface, under deformation by a pinning rod. Scale bar, 1 cm. **b**, Photograph of an A4 sheet of free-standing nanofilm after being transferred onto a water–air surface, where the arrows show the edge of the nanofilm. Scale bar, 5 cm. **c**, SEM surface and cross-sectional (inset) images of the nanofilm made from 0.1 wt% amino-functionalized β-cyclodextrin (β-CDA) and 0.1 wt% TPC reacted for 1 min (β-CDA-TPC-0.1) at the free interface and then transferred onto an alumina support. Scale bars, 1 μm (inset) and 500 nm (main). **d**, AFM height image of the nanofilm (β-CDA-TPC-0.01) on a silicon wafer. Scale bar, 200 nm. **e**, Height profile of the line scanned in **d**. **f**,**g**, GI-WAXS two-dimensional (**f**) and one-dimensional (**g**) images of the nanofilm (β-CDA-TPC-0.01), where *Q*_||_ denotes the reciprocal space parallel (||) to the nanofilm surface and *Q*_⊥_ denotes the reciprocal space perpendicular (⊥) to the nanofilm surface. a.u., arbitrary units. **h**, UHV AFM height images of the nanofilms incorporating small cavity (α-CDA-TPC-0.01), medium cavity (β-CDA-TPC-0.01) and large cavity (γ-CDA-TPC-0.01). Scale bars, 1 nm. **i**, Height profile of the lines scanned in **h**, where the lateral distances travelled between peaks show the pore widths. **j**, Pore size distributions for the nanofilm surfaces extracted from multiple UHV AFM samples of each macrocycle.

Nanofilms prepared at the free aqueous–organic interface were flexible and robust, with no sign of breaking or tearing when deformed using a rod (Fig. 2a). No film was observed if unfunctionalized macrocycles were used instead at the same conditions (Supplementary Fig. 24). The mechanical strength was maintained when scaling the nanofilm up to a larger area. Figure 2b shows a free-standing nanofilm of A4 sheet size (21 × 29.7 cm^2^) transferred onto a water–air surface, while remaining macroscopically defect-free. This mechanical integrity enabled the transfer of free-standing nanofilms onto ultrafiltration supports to provide composite membranes. Considering the scale-up potential, membranes of A4 sheet size are suitable for use in plate-and-frame^18^ or envelope-type modules^3^. Free-standing nanofilms have been fabricated continuously through dual layer slot coating^19^ or in-situ free interfacial polymerization^20^, enabling them to be wound into spiral modules for industrial applications.

Surface and cross-sectional scanning electron microscopy (SEM) images show a composite membrane comprising a nanofilm prepared from 0.1 wt% amino-functionalized β-cyclodextrin (β-CDA) and 0.1 wt% TPC reacted for 1 min (β-CDA-TPC-0.1) at an aqueous–organic interface and then transferred onto an alumina support (Fig. 2c). By manipulating the concentration, the nanofilm thickness could be reliably controlled (Supplementary Figs. 25–29). Atomic force microscopy (AFM) showed the thickness of the nanofilm (β-CDA-TPC-0.01) to be approxmately 3.5 nm (Fig. 2d,e), equivalent to three aligned cyclodextrin units. This is 30 times thinner than the polyester layers created from randomly packed cyclodextrins^7^.

## Aligned macrocycles create pores

Grazing-incidence wide-angle X-ray scattering (GI-WAXS) confirmed that following polymerization a crystalline nanofilm (β-CDA-TPC-0.01) with a high degree of preferential orientation was formed. We note that the real-space location of the presumably low index diffraction features matches well with the expected size of the macrocycles (Fig. 2f). Moreover, the extracted one-dimensional X-ray scattering profile shows peaks at *Q*_||_ = 1.20 and *Q*_⊥_ = 1.74, 1.88 Å^−1^ (Fig. 2g), consistent with the channels formed by the macrocycle pores being aligned normal to the supporting substrate. The location and relative intensity of the interpolated linear X-ray scattering pattern is consistent with predominantly eclipse-stacked macrocycles between adjacent layers. The distribution of the intensity in these GI-WAXS patterns is not centred on a single point, but is found as a preferentially distributed arc of intensity. This indicates that whereas the macrocycles are predominantly aligned within the membrane, some distribution of this alignment is observed. This is expected because of the inherent flexibility of soft materials containing bonds that can be formed with a variety of conformations^21^.

Preferential alignment provides macrocycle pores facing upright towards the nanofilm surface, and enables visualization of their pore geometry. Ultra-high vacuum (UHV) AFM shows subnanometre pores in the surfaces of nanofilms incorporating aligned amino-functionalized cyclodextrins, α-CDA, β-CDA and γ-CDA (Fig. 2h). For example, the lateral distances travelled along the profile lines in Fig. 2h reflect pore widths of approximately 0.6 nm for the medium cavity β-CDA (Fig. 2i), which corresponds well to the theoretical upper rim diameter of 0.61 nm (Supplementary Table 2). There are more than 200 pores marked on this nanofilm surface (Supplementary Fig. 30), equivalent to roughly 60% porosity on the 100 nm^2^ area scanned, considering an average pore size of 0.6 nm. AFM profiles from multiple nanofilms of each macrocycle were collected to statistically analyse the pore size distribution, which shifts in an ascending order, in line with the increasing cavity size of cyclodextrins (Fig. 2j). Visualization of subnanometre pores has presented a challenge in verifying existence of porous structures and hence explaining the transport mechanism in polymer membranes. A key obstacle has been the deformation and collapse of ill-defined pores in amorphous polymer films under the vacuum conditions required for many microscopy techniques^6,22^. Here, the rigid structure of macrocycles and their ordered orientation ensures conservation of their well-defined cavities even under an UHV, enabling the pores to be visualized.

## Accurate molecular sieving

These results indicate that macrocycles have been polymerized regioselectively as a 3–4-macrocycle-thick film in an ordered orientation. We propose that this alignment of macrocycle cavities provides direct channels across the nanofilm with low tortuosity for solute diffusion. Solutes with dimensions smaller than but still close to the cavity size can permeate through these direct channels, whereas these molecules would be retained in nanofilms comprising disordered macrocycles due to hindered transport^23^. To explore this hypothesis, we manipulate the macrocycle orientation by altering the crosslinker chemistry, flipping the nanofilm surface and controlling the nanofilm thickness.

**Fig. 3:**
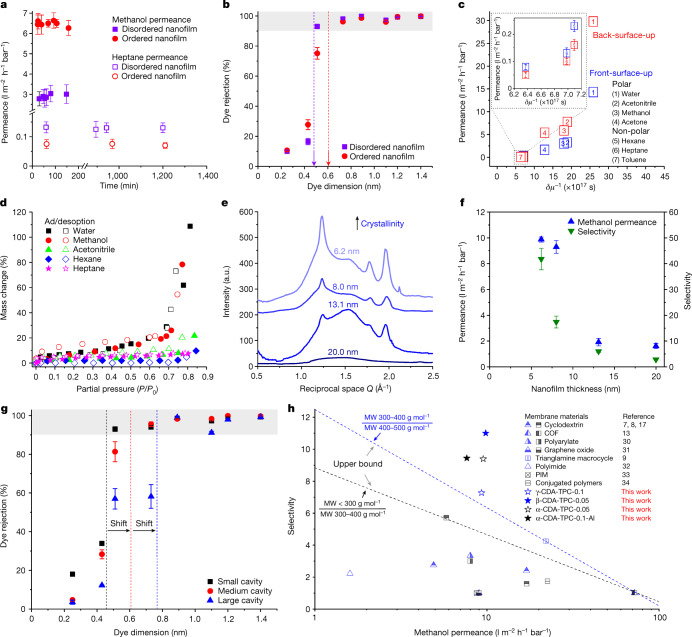
Performance of composite membranes comprising ultrathin polyamide nanofilms incorporating aligned macrocycle pores on PAN supports. **a**, Plot of methanol and heptane permeances over time for disordered nanofilm (β-CDA-TMC-0.1) and ordered nanofilm (β-CDA-TPC-0.1). **b**, Dye rejections of the disordered nanofilm and the ordered nanofilm, where the grey shadow indicates the cut-off rejections (≥90%) of dye molecules. **c**, Liquid permeances of the nanofilm (β-CDA-TMC-0.1) as a function of the solvent parameter ($\delta$) and the viscosity ($\mu$), where front-surface-up and back-surface-up indicate the active nanofilm surface facing the feed. **d**, Vapour sorption isotherm for nanofilms (β-CDA-TMC-0.1) in various solvents. **e**, GI-WAXS one-dimensional image. **f**, Methanol permeance and selectivity of the nanofilms made from γ-CDA-TPC with varying thickness. **g**, Dye rejections of the nanofilms incorporating small cavity (α-CDA-TPC-0.05), medium cavity (β-CDA-TPC-0.05) and large cavity (γ-CDA-TPC-0.05) with increasing cavity/molecular size. **h**, Trade-off between methanol permeance and selectivity between solutes of different molecular weights (MW) for membranes reported in literature and fabricated in this work. Upper bound lines were added to guide the reader. All experiments were carried out in a dead-end filtration cell under pressure of 10 bar at 25 °C with constant stirring of 250 r.p.m. The error bars represent reproducible experiments for at least three independent membrane samples.

An alternative crosslinker, trimesoyl chloride (TMC), was used to prepare disordered nanofilm β-CDA-TMC-0.1, which showed no Bragg diffraction peaks, in contrast to concentrated scattering density in the ordered nanofilm β-CDA-TPC-0.1 (Supplementary Fig. 31). Both nanofilms were transferred onto polyacrylonitrile (PAN) supports to form composite membranes used for OSN (Supplementary Figs. 32 and 33). The ordered nanofilm provides double the methanol permeance of the disordered nanofilm, whereas the trend reverses for heptane (Fig. 3a). As both nanofilms showed similar thickness and crosslinking degree (Supplementary Figs. 25, 26 and 34), we anticipate that permeance differences can be attributed to the orientation of macrocycles. Randomly orientated macrocycles preferentially expose their hydrophobic walls, rather than the hydrophilic rims, to the nanofilm surface (Supplementary Fig. 35), resulting in slower transport of polar solvent methanol. A range of dyes was dissolved in methanol to investigate the molecular sieving performance of these membranes (Supplementary Table 3). Disordered nanofilms show a cut-off rejection (≥90%) at 0.48 nm (Fig. 3b), which deviates from the pore size of β-CDA (0.61 nm). The anomalously high rejection of small dyes is attributed to increased hindered diffusion through disordered nanofilms^23^. By contrast, ordered nanofilms showed precise sieving consistent with the β-CDA cavity size. When a ternary feed containing two dyes in methanol was filtered through ordered nanofilms, similar rejections and hence selectivity were observed (Supplementary Fig. 36).

To further demonstrate the impact of orientation, free-standing nanofilms were flipped over to expose their back surfaces to the feed^24^. Both back-surface-up and front-surface-up nanofilms showed fast transport of polar solvents (Fig. 3c). This agrees with the chemical affinity of the solvents (Supplementary Table 4), for which the nanofilm provided an order of magnitude higher vapour adsorption in polar solvents than non-polar solvents (Fig. 3d). Back-surface-up nanofilms showed double the permeances for polar solvents compared to front-surface-up nanofilms without compromising the solute rejections (Supplementary Table 5), whereas the trend reversed for non-polar solvents (Fig. 3c). This is attributed to the chemical heterogeneity of these nanofilms. Back-surface-up nanofilms are rich in hydrophilic and negatively charged hydroxyl groups (Supplementary Figs. 35 and 37), thereby enhancing transport of polar solvents. By contrast, surface-independent permeances are observed in chemically homogeneous polyamide nanofilms^24^.

We assert that the ultrathin nanofilm thickness is critical to packing the macrocycles in an ordered orientation. GI-WAXS demonstrated that the crystallinity increased as the thickness of the nanofilms incorporating γ-CDA reduced from 20.0 to 6.2 nm (Fig. 3e), indicating an enhanced alignment of macrocycles. Ultrathin nanofilm thickness restricts the orientation of macrocycles to preferentially packing in more ordered arrays, showing concentrated scattering intensity, whereas thicker nanofilms allow more freedom for macrocycles to pack randomly and show no X-ray diffraction features (Supplementary Figs. 38 and 39). Therefore, macrocycle cavities are aligned in ultrathin nanofilms to create subnanometre channels that provide faster solvent transport and precise molecular sieving corresponding to the cavity size (Supplementary Fig. 40), and hence higher solute selectivity than thicker nanofilms (Fig. 3f).

Polymerizing predefined macrocycles can manipulate pore diameter by using macrocycles of different cavity sizes. Amino-functionalized cyclodextrins with increasing cavity sizes were used to fabricate aligned macrocycle membranes on PAN supports. Methanol permeance increased as the cavity size increased (Supplementary Fig. 41). Membranes directly polymerized on PAN supports by means of conventional interfacial polymerization provided notably lower permeance and selectivity (Supplementary Fig. 42) due to the formation of thick separating layers (Supplementary Fig. 43). This may be because of interfacial instabilities caused by the exothermic crosslinking reaction, in which the heat released could not dissipate efficiently through the support membranes owing to their poor heat transfer^2^. Herein, a key advantage of fabrication at a free interface is the efficient heat dissipation through the bulk water solution, in the absence of ultrafiltration supports^24^. This enables fabrication of ultrathin nanofilms an order of magnitude thinner than those prepared on supports^7–9,17^, and allows alignment of macrocycle cavities into permeation channels. The permeance can be further improved to 11.9 ± 0.4 l m^−2^ h^−1^ bar^−1^ by transferring nanofilms onto less compressible alumina supports (β-CDA-TPC-0.1-Al, Supplementary Figs. 44–46), which doubles the methanol permeance of composite membranes incorporating randomly packed cyclodextrins^7,8^. Using these nanofilms for dye separations, the changes in cut-off rejections corresponded well to the cavity sizes of cyclodextrins (Fig. 3g). Similar behaviour was observed for other amino-functionalized macrocycles (for example, SC[4]AA, Supplementary Figs. 47 and 48). Overall, the macrocycle cavities were translated into membrane pores giving ångström-scale differentiation between 0.4 and 0.8 nm.

## High-value pharmaceutical separations

This creates potential for applying aligned macrocycle membranes in high-value pharmaceutical separations requiring accurate molecular sieving, exemplified here by the enrichment of cannabidiol oil (CBD). The demand for production of CBD has grown rapidly owing to its efficacy in treatment of anxiety, depression and cancer^25^, with a forecast global market of US$2 billion by 2022 (ref. ^26^). Current state-of-the-art processes for producing CBD rely on extraction and chromatography^27,28^, which are expensive and energy intensive. Recently, membranes have been advanced as an alternative for purification and enrichment of CBD from hemp oil by means of OSN^29^. Critical to this opportunity is accurate differentiation of CBD from other solutes of similar dimensions dissolved in the extraction solvent ethanol (Supplementary Fig. 49). The extract comprises three primary classes of molecules: large molecules such as chlorophyll and β-carotene (>400 g mol^−1^), CBD and derivatives (300–400 g mol^−1^), and limonene and other smaller molecules (<300 g mol^−1^). Therefore, sharp selectivity between these molecular weight domains is the key for successful separation. Compared to commercially available polyamide nanofiltration membranes and state-of-the-art research membranes reported in literature^7–9,13,17,30–34^, aligned macrocycle membranes showed high selectivity in this target range (Fig. 3h and Supplementary Tables 5 and 6), making them a competitive candidate for enriching CBD.

**Fig. 4:**
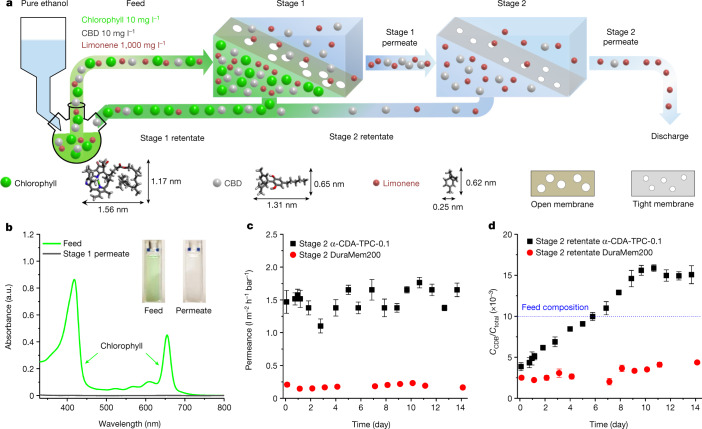
Separation of CBD by using ultrathin polyamide nanofilms incorporating aligned macrocycle pores. **a**, Schematic demonstrating the cascade membrane process for enriching CBD from a synthetic quaternary feed containing limonene, CBD and chlorophyll in ethanol, where Stage 1 separates chlorophyll from the mixture using an open membrane and Stage 2 enriches CBD using a tight membrane. The process was carried out in a continuous crossflow system under 10 bar transmembrane pressure. **b**, UV–vis absorption spectra of chlorophyll in the feed and permeate from Stage 1 for an open membrane incorporating aligned large macrocycle pores (γ-CDA-TPC-0.1). The inset shows photographs of feed and permeate. **c**, Ethanol permeance over time for nanofilms incorporating aligned small macrocycle pores (α-CDA-TPC-0.1) and commercial standard membrane DuraMem200 used for enriching CBD. **d**, The concentration of CBD (*C*_CBD_) against the concentration of all solutes (*C*_Total_) in Stage 2 retentate over time for aligned macrocycle membranes (α-CDA-TPC-0.1) versus commercial membranes. The error bars represent reproducible experiments for at least three independent membrane samples.

Two membranes in a cascade process are required for enrichment of CBD: an open one that permeates CBD and limonene, and a tight one that permeates limonene alone and enriches CBD (Fig. 4a and Supplementary Fig. 50). Macrocycles with large and small cavities were used to fabricate open and tight membranes, whereas commercial membranes DuraMem500 and DuraMem200, the current benchmark used for CBD purification, were deployed as counterparts at each stage^29^. At Stage 1, membranes of aligned large macrocycles (γ-CDA-TPC-0.1) allowed passage of 15% CBD and but only <0.1% chlorophyll (Fig. 4b), which transported 6% more CBD than DuraMem500 membranes (Supplementary Fig. 51). At Stage 2, membranes of aligned small macrocycles (α-CDA-TPC-0.1) showed one order of magnitude higher ethanol permeance than DuraMem200 membranes (Fig. 4c). Moreover, the well-ordered α-CDA-TPC-0.1 nanofilms concentrated CBD after 7 days and eventually achieved 50% enrichment (Fig. 4d). By comparison, commercial membranes DuraMem200 achieved only one-third of the CBD concentration in the same timeframe.

Whereas much research focuses on permeance, we assert that membranes with high selectivity between molecules are more pressing for energy-intensive and high-value industries requiring separations^35,36^. Here, we fabricate ultrathin nanofilms incorporating aligned macrocycles and exploit their cavities to create subnanometre channels for separating molecules of close dimensions. The pore size is controlled at ångström precision corresponding to the macrocycle cavity size, enabling fast solvent transport and high selectivity in enriching CBD. Our work provides a feasible strategy to translate intrinsic cavities/apertures of porous materials into well-defined pores in polymer membranes, extending their potential use into processes requiring accurate molecular selectivity.

## Methods

### Chemicals used

α-cyclodextrin (α-CD) (≥98%, Sigma-Aldrich), β-cyclodextrin (β-CD) (≥97%, Sigma-Aldrich), γ-cyclodextrin (γ-CD) (≥98%, Sigma-Aldrich), 4-sulfocalix[4]arene sodium salt (SC[4]A) (≥98%, Tokyo Chemical Industry Ltd), ethylenediamine (EDA) (ReagentPlus, ≥99%, Sigma-Aldrich), 1,1′-carbonyldiimidazole (CDI) (≥97%, Sigma-Aldrich), TPC (≥98%, Sigma-Aldrich) and TMC (≥98%, Sigma-Aldrich) were used as received without further purification. Pure chlorophyll a and cannabidiol (CBD) solution (10 mg ml^−1^ in ethanol) were purchased from Sigma-Aldrich. (+)-Limonene (>99%) was purchased from Tokyo Chemical Industry Ltd. Single crystal silicon wafers (phosphorous doped, (100) polished) from Si-Mat Germany were used as a substrate to deposit the free-standing nanofilms for AFM measurement. PLATYPUS silicon wafers with 100-nm-thick gold coating from Agar Scientific were used to deposit the free-standing nanofilms for X-ray photoemission spectroscopy (XPS) measurements. PAN (230,000 g mol^−1^) powder was obtained from Goodfellow. All solvents used for phase inversion, interfacial polymerization and nanofiltration experiments were purchased from VWR. Commercial membranes DuraMem500 and DuraMem200 manufactured by Evonik were purchased from Sterlitech.

### Characterization methods

#### NMR

^1^H NMR, ^13^C NMR, ^13^C DEPT-135 NMR, ^1^H-^1^H 2D-COSY NMR and ^1^H-^13^C 2D-HSQC NMR spectra were recorded on a Brüker AVANCE III-400 spectrometer, with working frequencies of 400 (^1^H) and 101 (^13^C) MHz using deuterium oxide (D_2_O) as a solvent at 293 K (ref. ^16^). Chemical shift of D_2_O is given in ppm relative to the signal corresponding to the residual H_2_O: D_2_O, *δ*_H_ = 4.80 ppm. Note that before the NMR analysis, all solutions were passed through a pipette filled with cotton to remove the insoluble impurities and dust.

#### GI-WAXS

All GI-WAXS data were collected at Sector 8 of the Advanced Photon Source Argonne National Laboratory with a photon energy of 11 keV (*λ* = 1.127 Å)^21^. Samples were prepared by transferring the nanofilms onto silicon substrates with a native oxide layer. Before measurement, all samples were placed under vacuum to remove atmospheric scatter. All patterns were collected with an incident angle of *α* = 0.14°. Frames were taken on a Pilatus 1M camera. The exposure time and amount of attenuation was tuned to provide 80% maximum saturation of any pixel on the Pilatus detector. Radial linecuts of the data were collected by radially integrating along the Q axis using GIXSGUI.

#### UHV AFM

UHV AFM images were acquired by using a RHK UHV 7500 system at 5 × 10^−11^ mbar with a R9 controller^22^. Amplitude modulated tapping mode was operated at a temperature of 93 K using a liquid nitrogen flow cryostat. The nanofilms were annealed in UHV at 348 K for 30 min before the AFM measurements. AFM tips were sputtered with Ar^+^ ions at 680 eV for 90 s.

#### SEM

High resolution SEM (LEO 1525, Karl Zeiss) was used to characterize the surface and cross-sectional images of the nanofilms^24^. The nanofilms were sputtered with 15-nm-thick chromium coating (Q150T turbo-pumped sputter coater, Quorum Technologies Ltd) under an argon atmosphere (2 × 10^−2^ mbar).

#### AFM

The thickness of the nanofilm was measured using Multimode 8 (Bruker) atomic force microscope with E type scanner^24^. Free-standing nanofilms were transferred onto silicon wafers and dried at room temperature. A scratch was made to expose the wafer surface, so the height difference between the silicon wafer surface and the nanofilm surface reveals the nanofilm thickness. A resolution of 512 points per line was used. Gwyddion 2.44 SPM software was used to process the AFM images.

#### Water contact angle

Water contact angle was measured by the KRÜSS drop shape analyser. Before the test, each membrane sample was thoroughly rinsed with hexane to remove the residual acyl chloride, followed by air drying at room temperature overnight. A syringe with a blunt end dispensing tip was used to deliver the water droplet onto the membrane surface. The contact angle was continuously recorded for 1 min using a digital camera.

#### XPS

Oxford Materials Characterization Service and BegbrokeNano, Department of Materials, Oxford University provided the XPS measurements. Free-standing nanofilms were transferred onto a PLATYPUS gold coated silicon wafer, followed by air drying at room temperature. For each sample, at least three different spots with a size of 400 × 400 µm^2^ were scanned for the survey spectra and core level XPS spectra. The measurement was carried out in an ion pumped VG Microtech CLAM 4 MCD analyser system using 250-W monochromatic Al Kα (1,468.68 eV) excitation. Constant pass energy of 200 eV for wide scans and 20 eV for detailed scans were used^24^. To minimize sample charging, the C1s peak at BE 285 eV was used. Data were recorded using SPECTRA v.8 operating system, and processed by CasaXps. While measuring peak areas, background was subtracted by following the methods of Shirley^2^. C1s narrow scan spectra were deconvoluted into several characteristic peaks.

#### Zeta potential measurement at the membrane surface

Zeta potential of the membrane surface was measured by SurPASS zeta potential analyser from Anton Paar Ltd. For each test, two membranes were cut into 1 × 1 cm^2^ sheets and attached onto the holders with water-proof double-sided tape, followed by fixing the holders into the rectangular clamp cell with membrane surfaces facing to each other. The pH and conductivity were calibrated before the test. The system was washed thoroughly with deionized water before each test, followed by rinsing with an electrode solution consisting of 0.1 mM KCl. The pH was varied from 4 to 11 by using 50 mM HCl and NaOH solutions through titration, while the zeta potential of the surface was measured^24^.

#### Intelligent gravimetric analysis (IGA)

An IGA (IGA-002, Hiden Isochema) was used to perform the solvent vapour sorption experiments. Nanofilm powders were fabricated by mixing an aqueous solution containing 0.1 wt% amino-functionalized β-cyclodextrin with a hexane solution containing 0.1 wt% TMC under vigorous stirring for 1 min. The powders were subsequently filtered and thoroughly washed with methanol several times, and finally dried in vacuum oven at 70 °C overnight. Before each new isotherm, the nanofilm powder was heated to 100 °C under an UHV environment (1 × 10^−7^ mbar) until the sample mass was constant, ensuring the complete removal of residual solvent from previous experiments^37^. A minimum of 1 h was allowed at each pressure change in the isotherm experiment to reach steady state. The vapour pressure of each solvent studied was calculated at the operating temperature using the Antoine equation. The isotherm was carried out at a constant temperature of 25 °C. The mass change present in Fig. 3d was calculated as the mass of vapour adsorbed or desorbed over the mass of the dry nanofilm.

#### FTIR

FTIR spectra were recorded on a Perkin-Elmer Spectrum 100 spectrometer between wavenumbers of 4,000–500 cm^−1^. The instrument was equipped with a Universal ATR sampling accessory (diamond crystal), with a red laser excitation source (633 nm) and a middle infrared triglycine sulfate detector.

### Synthesis of amino-functionalized macrocycles

#### Amino-functionalized α-CD (α-CDA), 1

The dry α-CD (S1, 5.84 g, 6.0 mmol) and CDI (6.42 g, 39.6 mmol, 6.6 equiv.) were dissolved in anhydrous dimethyl sulfoxide (DMSO) (60 ml), and the resulting mixture was stirred under argon at room temperature for 12 h. Then excess EDA (60 ml, 900 mmol, 150 equiv.) was added, followed by continuous stirring for another 12 h. The resulting reaction solution was concentrated to 40 ml in vacuo, and precipitated in 500 ml of acetone and then filtrated off. The precipitate was redissolved in 40 ml of deionized water and reprecipitated in acetone (500 ml), and then the precipitate was filtered off and rinsed with acetone three times. The resulting precipitate was collected and dried to give the title compound as white powder (8.65 g, 96.8%).

^1^H NMR (400 MHz, D_2_O) *δ*_H_ = 4.86 (d, *J* = 3.56 Hz, 6H), 4.03–3.60 (m, 24H), 3.50–3.34 (m, 12H), 3.18–2.90 (m, 12H), 2.65–2.45 (m, 12H).

^13^C NMR (101 MHz, D_2_O) *δ*_C_ = 164.59 (6C, C=O), 101.43 (6C), 81.22 (6C), 73.30 (6C), 71.97 (6C), 71.60 (6C), 60.29 (6C), 40.10–39.64 (m, 12C, CH_2_CH_2_NH_2_).

#### Amino-functionalized β-CD (β-CDA), 2

The β-CDA was synthesized following a similar procedure to that described above. The β-CD (S2, 6.81 g, 6.0 mmol) and CDI (7.50 g, 46.2 mmol, 7.7 equiv.) were mixed in DMSO (60 ml), and stirred under argon at room temperature for 12 h. Then excess EDA (70 ml, 1,050 mmol, 175 equiv.) was added, followed by continuous stirring for another 12 h. The resulting mixture was concentrated to 40 ml under vacuum, precipitated in 500 ml of acetone and then filtered off. The precipitate was redissolved in 40 ml of deionized water and reprecipitated in acetone (500 ml), and then the precipitate was filtered off and rinsed with acetone three times. The resulting precipitate was collected and dried to give the title compound as white powder (9.74 g, 93.4%).

^1^H NMR (400 MHz, D_2_O) *δ*_H_ = 4.88 (d, *J* = 3.69 Hz, 7H), 3.95–3.56 (m, 28H), 3.49–3.32 (m, 14H), 3.15–2.91 (m, 14H), 2.67–2.45 (m, 14H).

^13^C NMR (101 MHz, D_2_O) *δ*_C_ = 164.59 (7C, C=O), 102.07 (7C), 81.26 (7C), 73.23 (7C), 71.95 (14C), 60.09 (7C), 40.14–39.65 (m, 14C, CH_2_CH_2_NH_2_).

#### Amino-functionalized γ-CD (γ-CDA), 3

The synthesis procedure for γ-CDA was as follows: a DMSO solution (50 ml) of γ-CD (S3, 5.20 g, 4.0 mmol) and CDI (5.71 g, 35.2 mmol, 8.8 equiv.) was stirred under argon at room temperature for 12 h. Then excess EDA (53.6 ml, 800 mmol, 200 equiv.) was added, followed by continuous stirring for another 12 h. The resulting reaction mixture was concentrated to 40 ml in vacuo, and precipitated in 500 ml of acetone and then filtered off. The precipitate was redissolved in 40 ml of deionized water and reprecipitated in acetone (500 ml), and then the precipitate was filtered off and rinsed with acetone three times. The resulting precipitate was collected and dried to give the title compound as white powder (7.76 g, 97.7%).

^1^H NMR (400 MHz, D_2_O) *δ*_H_ = 4.91 (d, *J* = 3.98 Hz, 8H), 4.00–3.60 (m, 32H), 3.50–3.34 (m, 16H), 3.20–3.00 (m, 16H), 2.68–2.55 (m, 16H).

^13^C NMR (101 MHz, D_2_O) *δ*_C_ = 164.69 (8C, C=O), 101.73 (8C), 80.47 (8C), 72.91 (8C), 72.23 (8C), 71.79 (8C), 60.09 (8C), 40.19–39.51 (m, 16C, CH_2_CH_2_NH_2_).

#### Amino-functionalized 4-sulfocalix[4]arene (SC[4]AA), 4

Similar to the synthesis of α-CDA (**1**) described above, a mixture of 4-sulfocalix[4]arene (S4, 3.62 g, 4.0 mmol) and CDI (3.89 g, 24 mmol, 6.0 equiv.) in DMSO (40 ml) was stirred under argon at ambient temperature for 12 h. Subsequently, excess EDA (32.1 ml, 480 mmol, 120 equiv.) was added, and the resulting solution was stirred overnight. After stripping off excess EDA, the residue was precipitated in acetone (500 ml) and filtered off, and the precipitate was redissolved in deionized water (40 ml) followed by reprecipitation in acetone. Then the resulting precipitate was rinsed with acetone three times, and then collected and dried in vacuo to afford the title compound (**4**, 4.56 g, 91.2%) as white powder.

^1^H NMR (400 MHz, D_2_O) *δ*_H_ = 7.75 (s, 8H, CH on Ph), 2.47 (s, 8H, CH_2_CH_2_NH_2_), 2.40 (s, 8H, CH_2_CH_2_NH_2_).

^13^C NMR (101 MHz, D_2_O) *δ*_C_ = 166.14 (4C, C=O), 134.60 (8C, CH on Ph), 130.69 (8C, C on Ph), 123.25 (8C, C on Ph), 39.44 (4C, CH_2_CH_2_NH_2_), 38.61 (4C, CH_2_CH_2_NH_2_).

### Fabrication of PAN supports by means of phase inversion

PAN support membranes were cast using a continuous casting machine (Sepratek). The dope solution was prepared by dissolving 11 wt% PAN powder in a mixture of 44.5 wt% DMSO and 44.5 wt% 1,3-dioxolane, and stirred overnight at 75 °C. Before casting, the dope solution was filtered through a 41-µm filter (NY4104700, Merck) and subsequently through a 11-µm filter (NY1104700, Merck) using a nitrogen pressurized filtration cell (XX4004740, Merck) at pressures of up to 70 psi (ref. ^37^). The membrane was cast on to a polyethylene terephthalate non-woven fabric (Hirose RO grade). The gap between the casting knife and the backing was set at 120 µm. The casting speed was controlled by the winder tension at 4 r.p.m. After casting, the support was immediately immersed into a water bath at 60 °C for 3 h, followed by drying at room temperature.

### Fabrication of polyamide nanofilms at a free aqueous–organic interface

A free aqueous–organic interface was created between an aqueous phase containing amino-functionalized macrocycles and a hexane phase containing acyl chloride in a glassware container^24^. After a certain reaction time (1 min for amino-functionalized cyclodextrins and 20 min for amino-functionalized 4-sulfocalix[4]arene), nanofilms were picked up on a substrate and rinsed with excess hexane to remove residual acyl chloride, followed by floating them on a water surface. The nanofilms were then transferred onto PAN or alumina supports to incorporate them into thin film composite membranes for nanofiltration experiments, or onto other substrates for characterization.

### OSN in a dead-end cell for dye separations

OSN experiments for dye separations were carried out at 10 bar in a dead-end stirred cell (Sterlitech) at 25 °C and under a constant stirring speed of 250 r.p.m. At least three membranes were tested for each condition to confirm the reproducibility. The effective membrane area was 12.56 cm^2^. The permeance and rejection were measured after steady permeance was achieved. The concentration of feed, permeate and retentate solutions was measured by ultraviolet–visible (UV–vis) absorption. UV spectra were recorded on a UV-1800 Shimazdu spectrophotometer in the range of 200 to 800 nm. The concentration and hence the rejection was calculated on the basis of the absorption values at the characteristic wavelength of dyes. The permeance (*P*) was calculated as follows:

1 $$P=\frac{V}{A\times {\rm{\bigtriangleup }}t\times {\rm{\bigtriangleup }}P}$$

where $V$ is the volume of permeate collected (l), $A$ is the area of the membrane (m^2^), $\triangle t$ is the time elapsed for collecting the required permeate volume (h) and $\triangle P$ is the transmembrane pressure (bar). The unit of the permeance was litres per square metre per hour per bar (l m^−2^ h^−1^ bar^−1^), which is the conventional standard.

Selectivity was calculated as the concentration ratio of two dyes in the permeate to the feed. In particular for the nanofilm produced from γ-CDA (upper pore width 0.77 nm) and TPC, the two dyes with dimensions across the upper pore width of γ-CDA, Safranin O (small dye, 0.73 × 0.97 nm^2^) and congo red (big dye, 0.89 × 2.4 nm^2^), were used to calculate the selectivity. For trade-off between permeance and selectivity of molecular weight 300–400 g mol^−1^ against 400–500 g mol^−1^, methyl orange (small dye, 0.51 × 1.5 nm^2^, 327 g mol^−1^) and sunset yellow (big dye, 1.1 × 1.7 nm^2^, 452 g mol^−1^) were used to calculate the selectivity.

2 $${\rm{Selectivity}}=\frac{{C}_{{\rm{permeate}},{\rm{small}}}/{C}_{{\rm{feed}},{\rm{small}}}}{{C}_{{\rm{permeate}},{\rm{big}}}/{C}_{{\rm{feed}},{\rm{big}}}}$$

where ${C}_{{\rm{permeate}},{\rm{small}}}$ is the concentration of the small dye in the permeate, ${C}_{{\rm{feed}},{\rm{small}}}$ is the concentration of the small dye in the feed, ${C}_{{\rm{permeate}},{\rm{big}}}$ is the concentration of the big dye in the permeate and ${C}_{{\rm{feed}},{\rm{big}}}$ is the concentration of the big dye in the feed.

### Diafiltration in a cascade process for enriching CBD

Diafiltration experiments for enriching CBD were carried out in a cascade process with two stages^38,39^. Stage 1 comprised two cells in series containing membranes with open pores, and stage 2 comprised one cell containing a membrane with tight pores. The transmembrane pressure at each stage was maintained at 10 bar, and the temperature was kept constant at 25 °C. Before their use in this experiment, commercial membranes DuraMem200 and DuraMem500 were soaked in pure ethanol overnight to remove any conditioning preservatives. A synthetic mixture of feedstock solution comprising 10 mg l^−1^ chlorophyll a, 10 mg l^−1^ CBD and 1,000 mg l^−1^ limonene was prepared in ethanol. A high-performance liquid chromatography (HPLC) pump was used to introduce the feed solution to stage 1 at a flowrate of 20 ml min^−1^. A gear pump was used for each stage to circulate the solution around the stage at 100 l h^−1^. The permeate from stage 1 was circulated into stage 2 as the feed. The permeate of stage 2 was collected for analysis. The retentate of stage 1 and stage 2 was recycled into the feedstock solution. Pure ethanol was used to top up the feedstock and so maintain its volume throughout the experiment. Concentrations of chlorophyll were determined by UV absorption spectra. Agilent 1100 series HPLC with ACE UltraCore 5 SuperC18 column (250 × 4.6 mm) was used to analyse the concentrations of CBD and limonene. The HPLC mobile phases were prepared by dissolving 0.1% formic acid in water and acetonitrile, respectively^40^.

## Online content

Any methods, additional references, Nature Research reporting summaries, source data, extended data, supplementary information, acknowledgements, peer review information; details of author contributions and competing interests; and statements of data and code availability are available at 10.1038/s41586-022-05032-1.

## Supplementary information

Supplementary Information Supplementary Figs. 1–51, Tables 1–6 and references.

## Data Availability

The data that support the findings of this study are available from the corresponding author upon request.
