# Supplementary material for: Aligned macrocycle pores in ultrathin films for accurate molecular sieving
Source: Nature. 2022 Aug 31;609(7925):58–64. doi: 10.1038/s41586-022-05032-1 (PMC9433321; doi:10.1038/s41586-022-05032-1)
Supplement: Supplementary file 1 — Supplementary Figs. 1–51, Tables 1–6 and references. [file 41586_2022_5032_MOESM1_ESM.pdf]

nature portfolio

https://doi.org/10.1038/s41586-022-05032-1

Supplementary information

# Aligned macrocycle pores in ultrathin films for accurate molecular sieving

In the format provided by the authors and unedited

## *Supplementary Information*

# Aligned macrocycle pores in ultrathin films for accurate molecular sieving

Zhiwei Jiang[1,2,7], Ruijiao Dong[1,3,7], Austin M. Evans[4,5], Niklas Biere[6], Mahmood A. Ebrahim[1], Siyao Li[1], Dario Anselmetti[6], William R. Dichtel[4], Andrew G. Livingston[1,2]*

**Affiliations:**

[1]Barrer Centre, Department of Chemical Engineering, Imperial College London, South Kensington Campus, London SW7 2AZ, UK.

[2]Department of Engineering and Materials Science, Queen Mary University of London, Mile End Road, London E1 4NS, UK.

[3]Shanghai Centre for Systems Biomedicine, Key Laboratory of Systems Biomedicine (Ministry of Education), Shanghai Jiao Tong University, Shanghai, 200240, China.

[4]Department of Chemistry, Northwestern University, Evanston, IL 60208, USA.

[5]George & Josephine Butler Polymer Research Laboratory, Center for Macromolecular Science & Engineering, Department of Chemistry, University of Florida, Gainesville, FL 32611, USA.

[6]Experimental Biophysics and Applied Nanoscience, Faculty of Physics, Bielefeld University, 33615 Bielefeld, Germany.

[7]These authors contributed equally to this work.

*Correspondence to: a.livingston@qmul.ac.uk

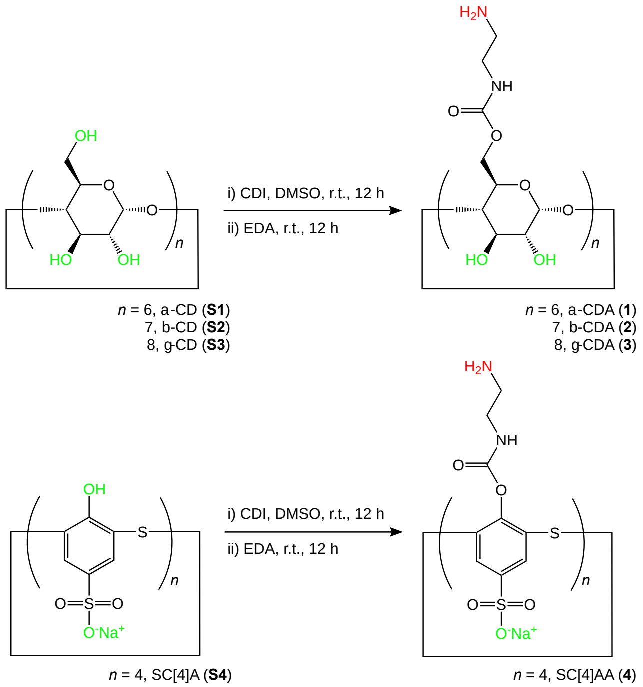


**Supplementary Figure 1 | Synthesis of amino-functionalised macrocyclic derivatives including $\alpha$-CDA (1), $\beta$-CDA (2), $\gamma$-CDA (3), and SC[4]AA (4).**

## NMR spectra

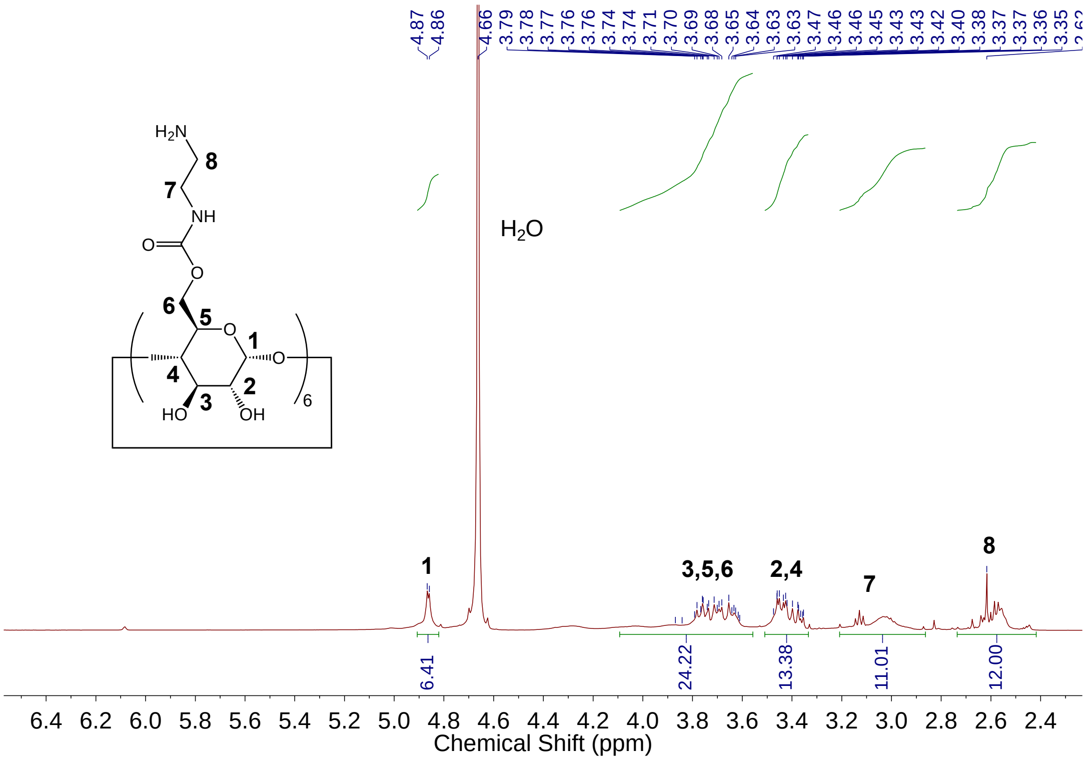


**Supplementary Figure 2 | $^1$H NMR spectrum of $\alpha$-CDA (1) in $D_2O$.**

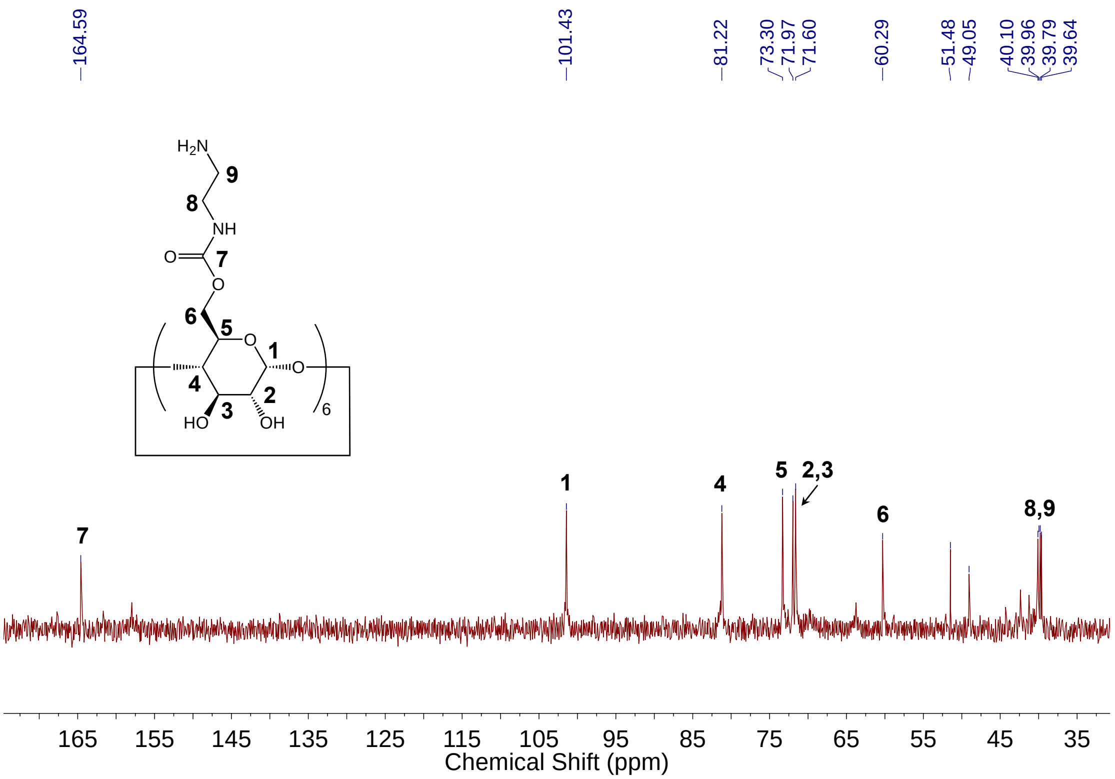


**Supplementary Figure 3 | $^{13}$C NMR spectrum of $\alpha$-CDA (1) in $D_2O$.**

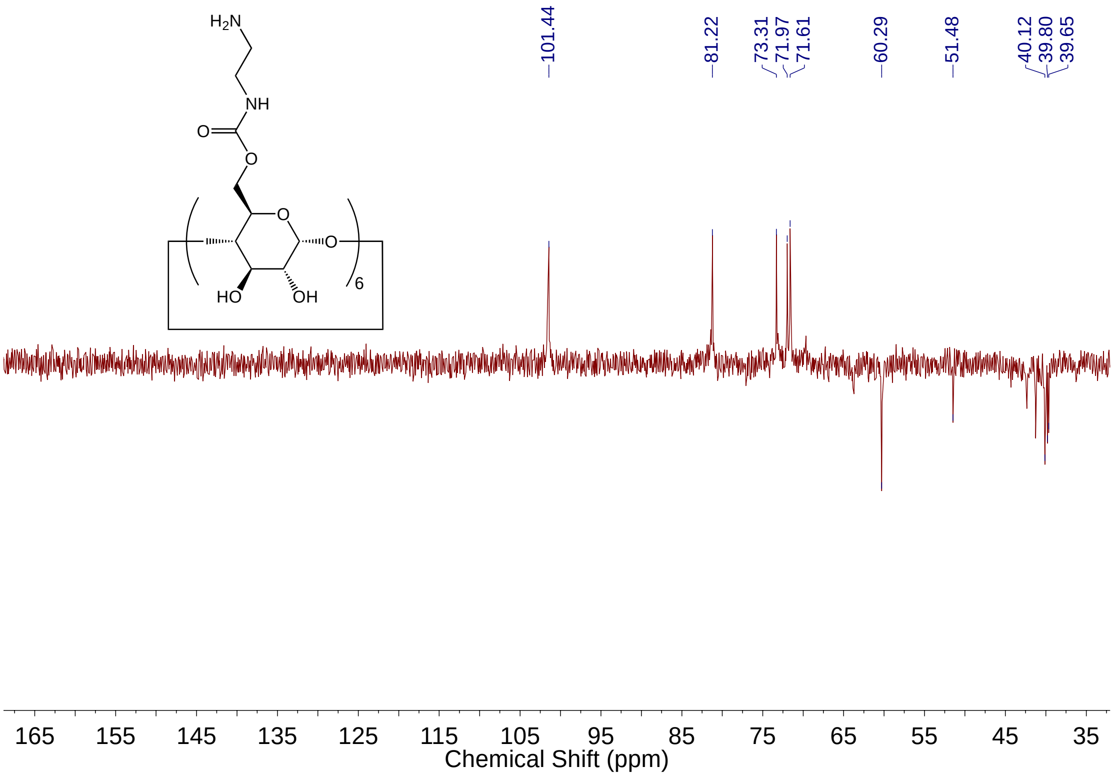


**Supplementary Figure 4 | $^{13}$C DEPT-135 NMR spectrum of $\alpha$-CDA (1) in $D_2O$.**

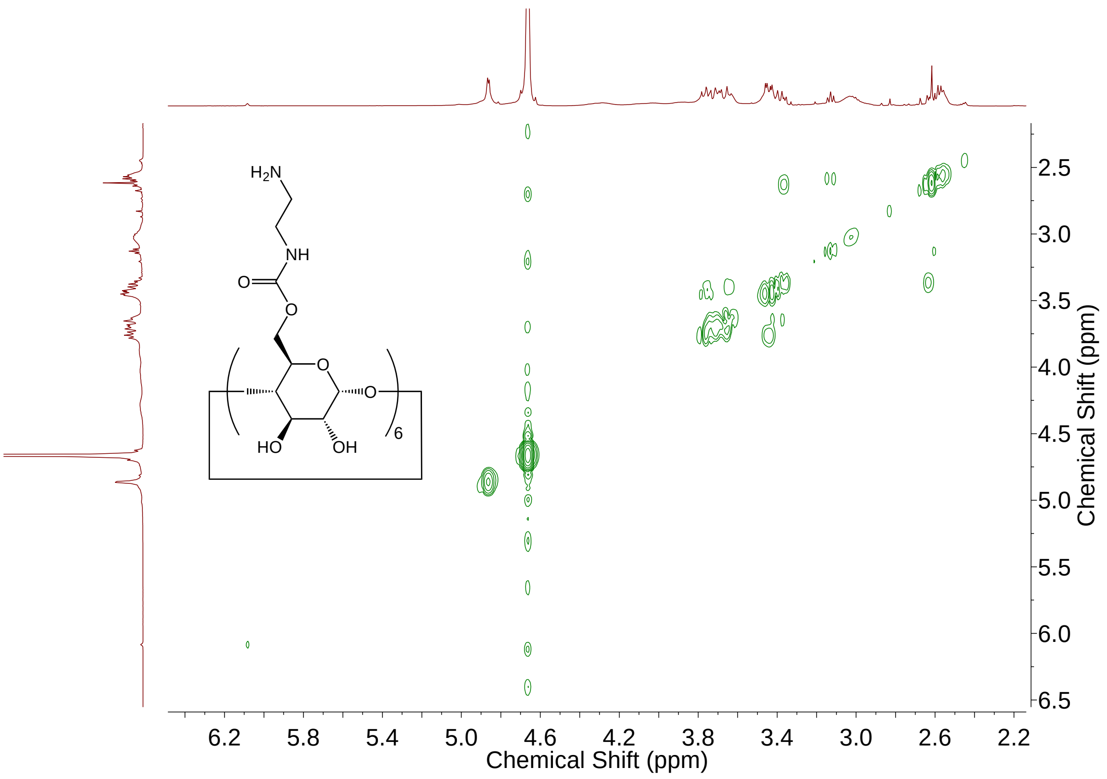


**Supplementary Figure 5 | 2D $^{1}H$-$^{1}H$ COSY NMR spectrum of $\alpha$-CDA (1) in $D_2O$.**

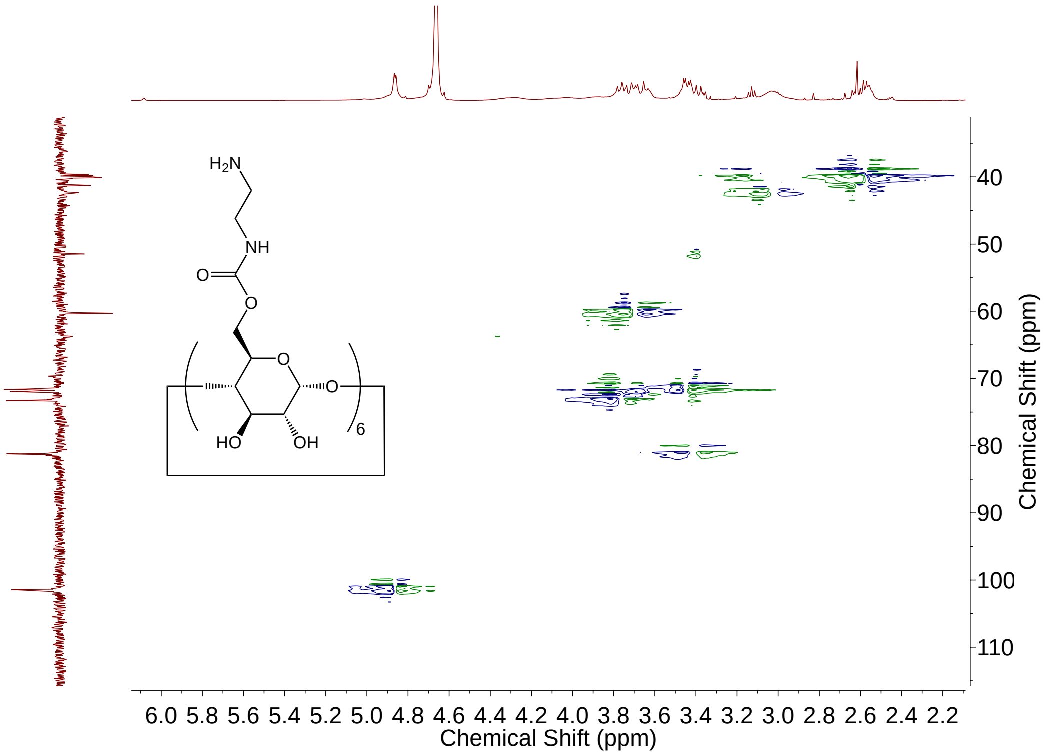


**Supplementary Figure 6 | 2D $^{1}H$-$^{13}C$ HSQC NMR spectrum of $\alpha$-CDA (1) in $D_2O$.**

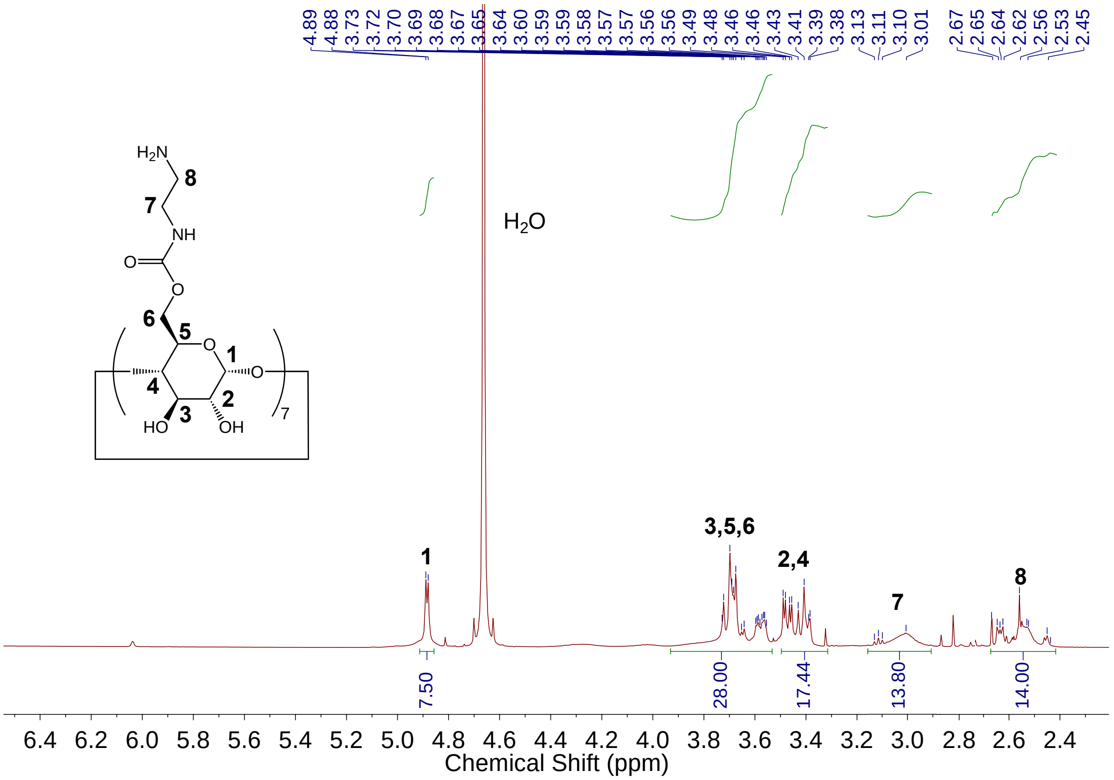


**Supplementary Figure 7 | $^{1}$H NMR spectrum of *β*-CDA (2) in $D_2O$.**

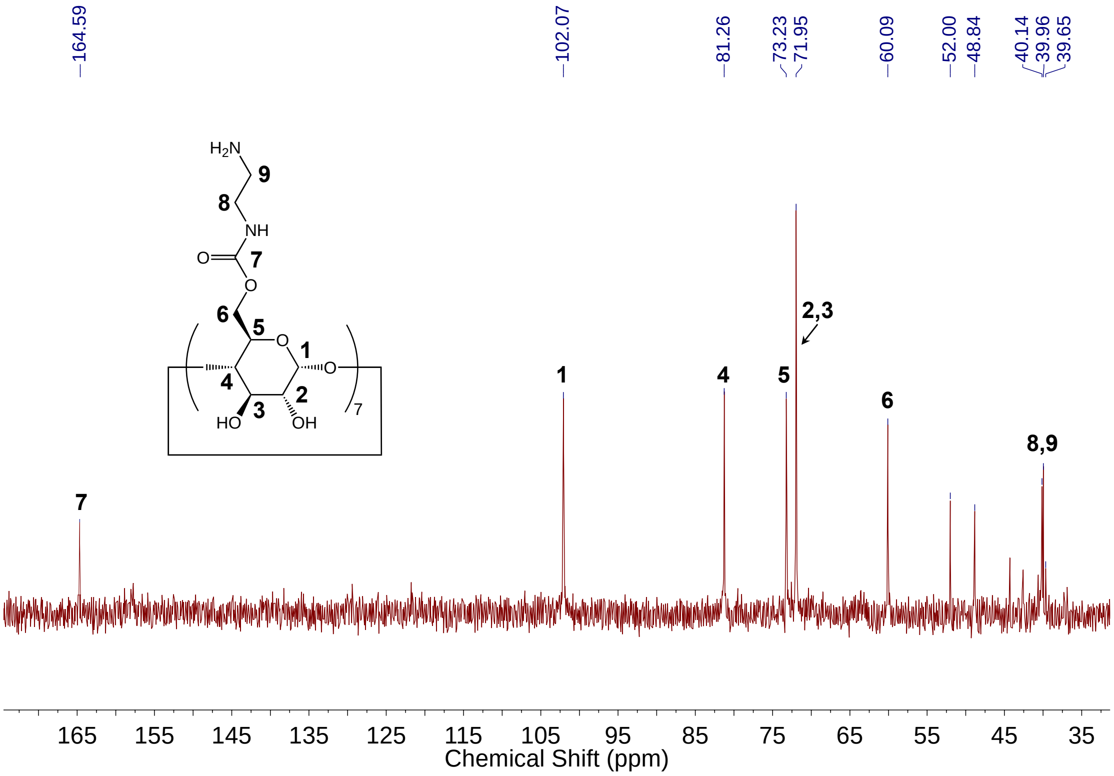


**Supplementary Figure 8 | $^{13}$C NMR spectrum of *β*-CDA (2) in $D_2O$.**

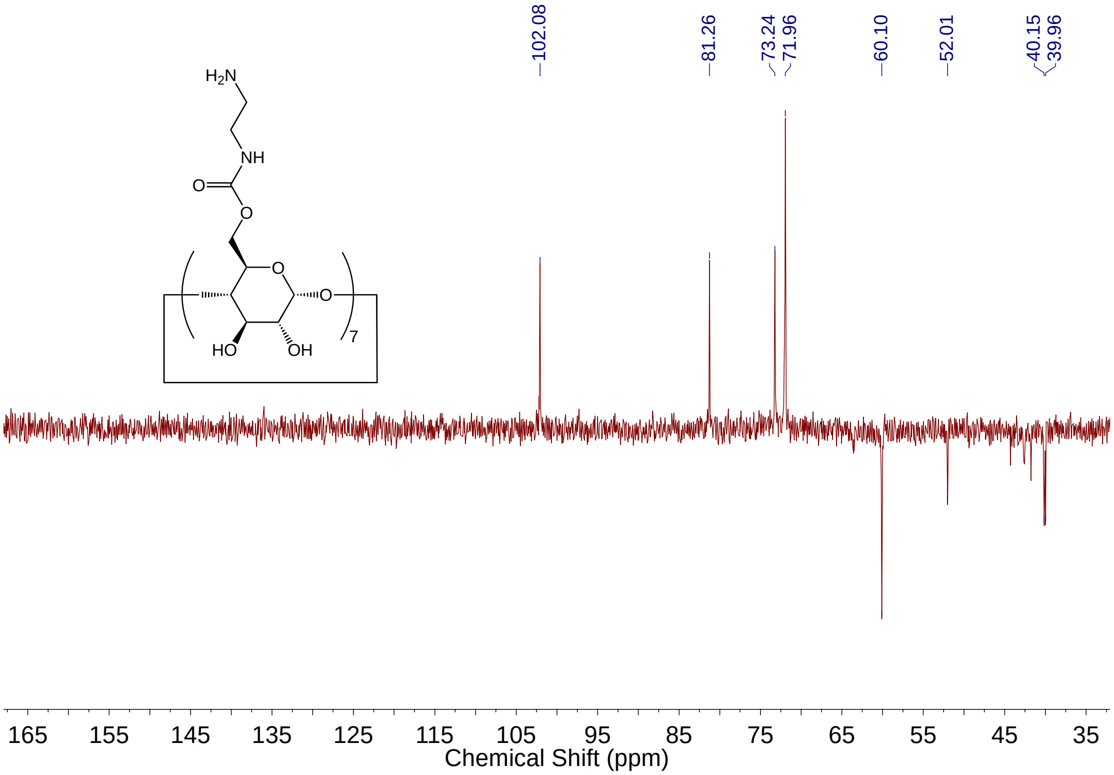


**Supplementary Figure 9 | $^{13}$C DEPT-135 NMR spectrum of *β*-CDA (2) in D$_2$O.**

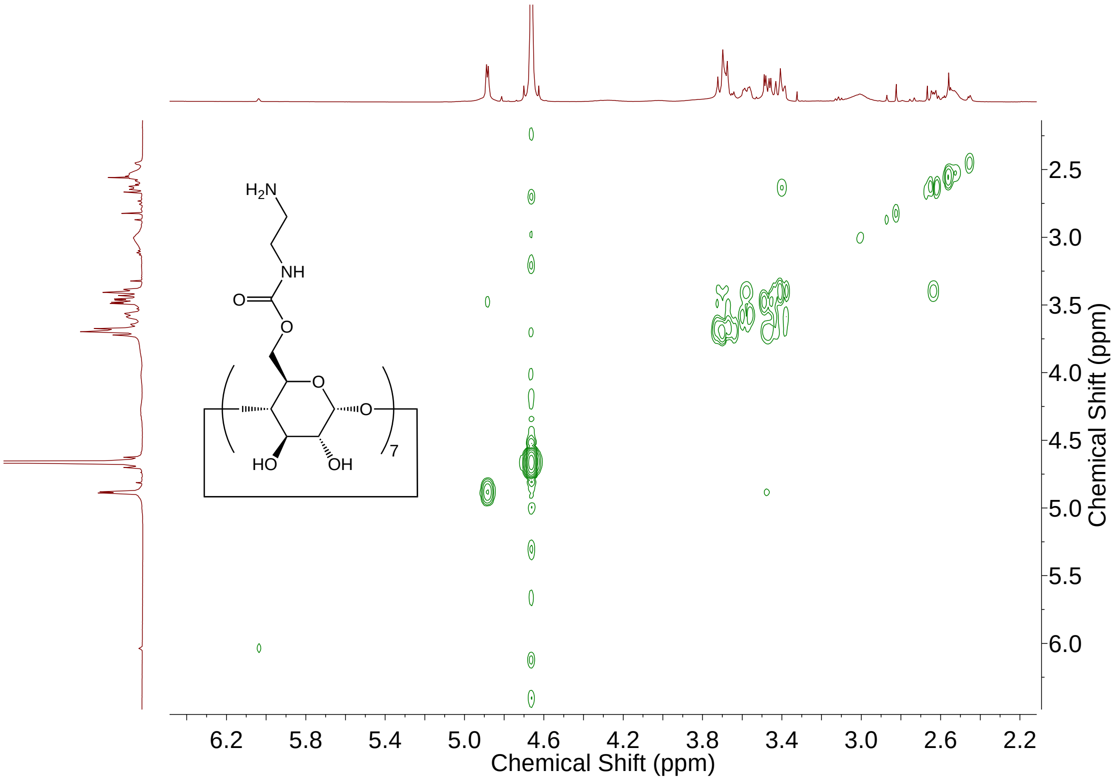


**Supplementary Figure 10 | 2D $^{1}$H-$^{1}$H COSY NMR spectrum of *β*-CDA (2) in D$_2$O.**

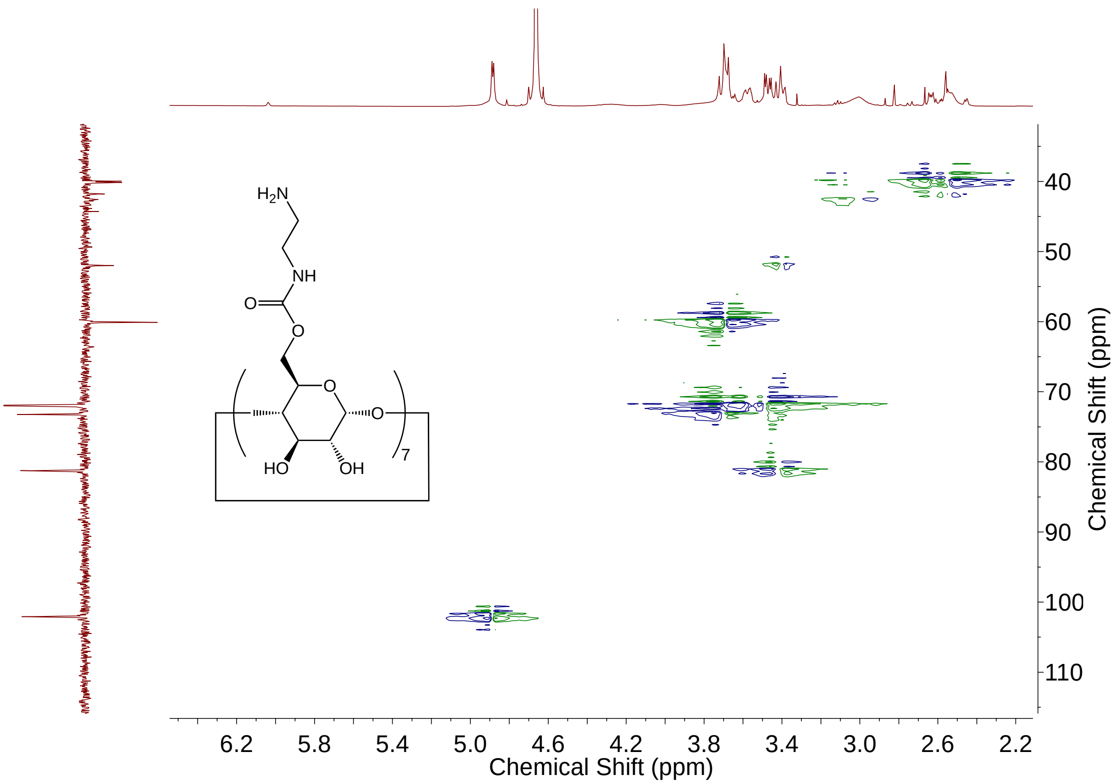


**Supplementary Figure 11 | 2D $^{1}H$-$^{13}C$ HSQC NMR spectrum of *β*-CDA (2) in $D_2O$.**

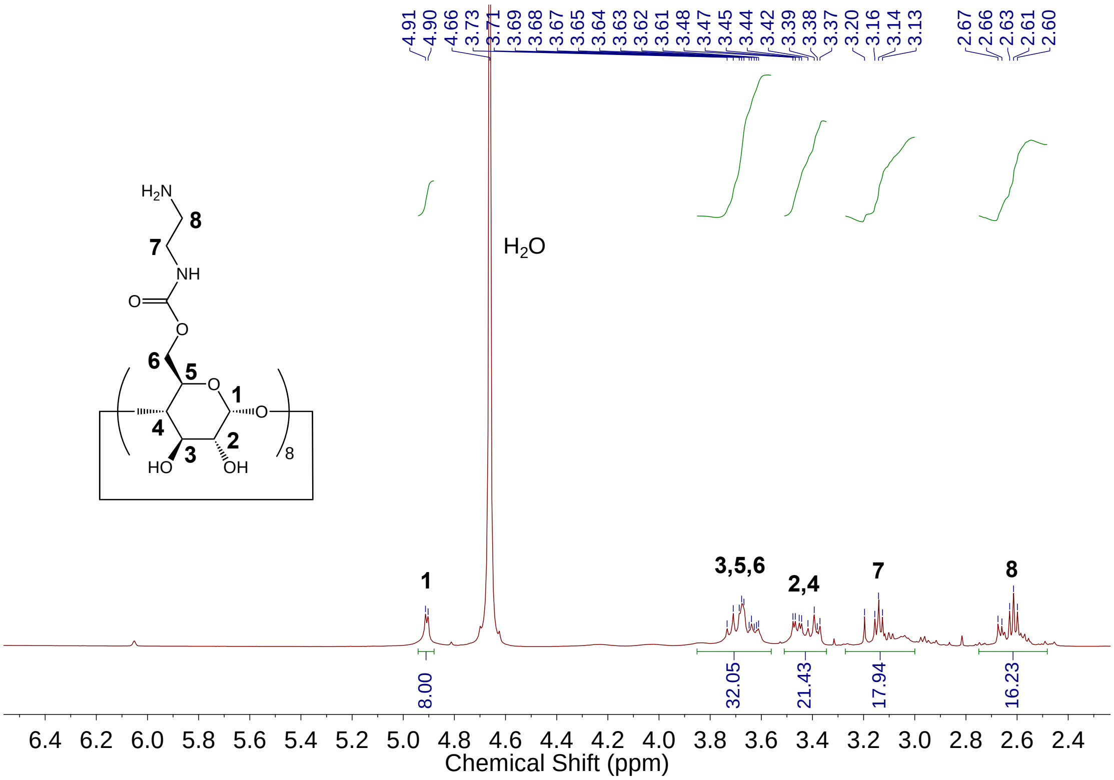


**Supplementary Figure 12 | $^{1}H$ NMR spectrum of *γ*-CDA (3) in $D_2O$.**

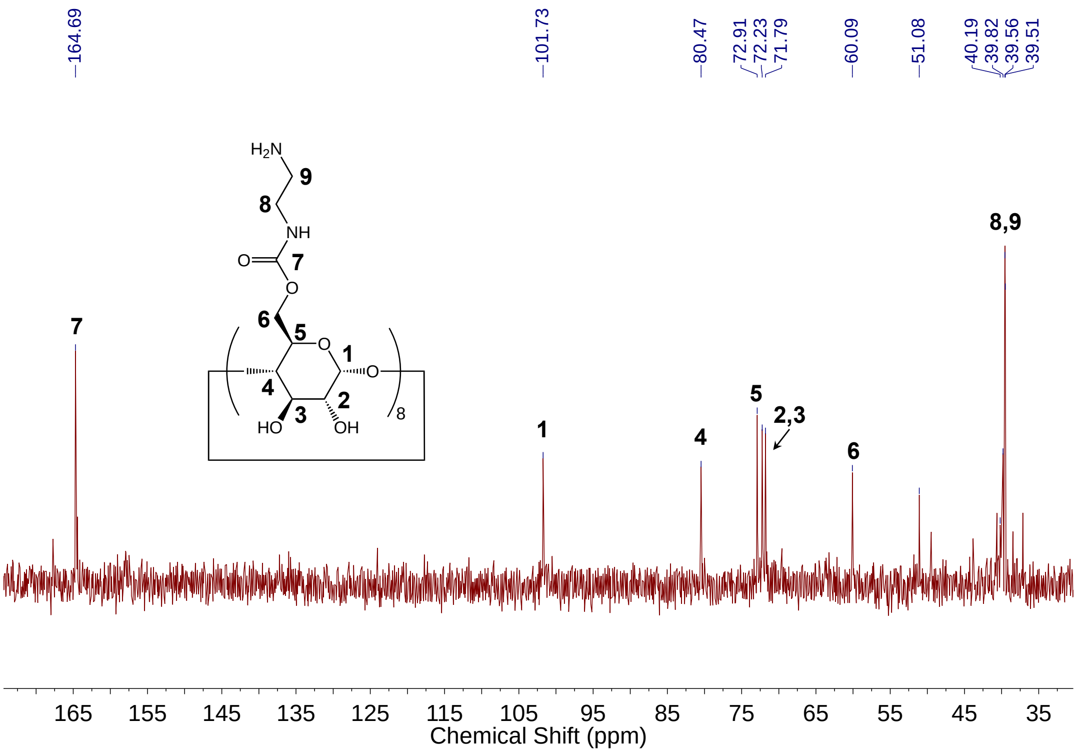


**Supplementary Figure 13 | $^{13}$C NMR spectrum of *γ*-CDA (3) in $D_2O$.**

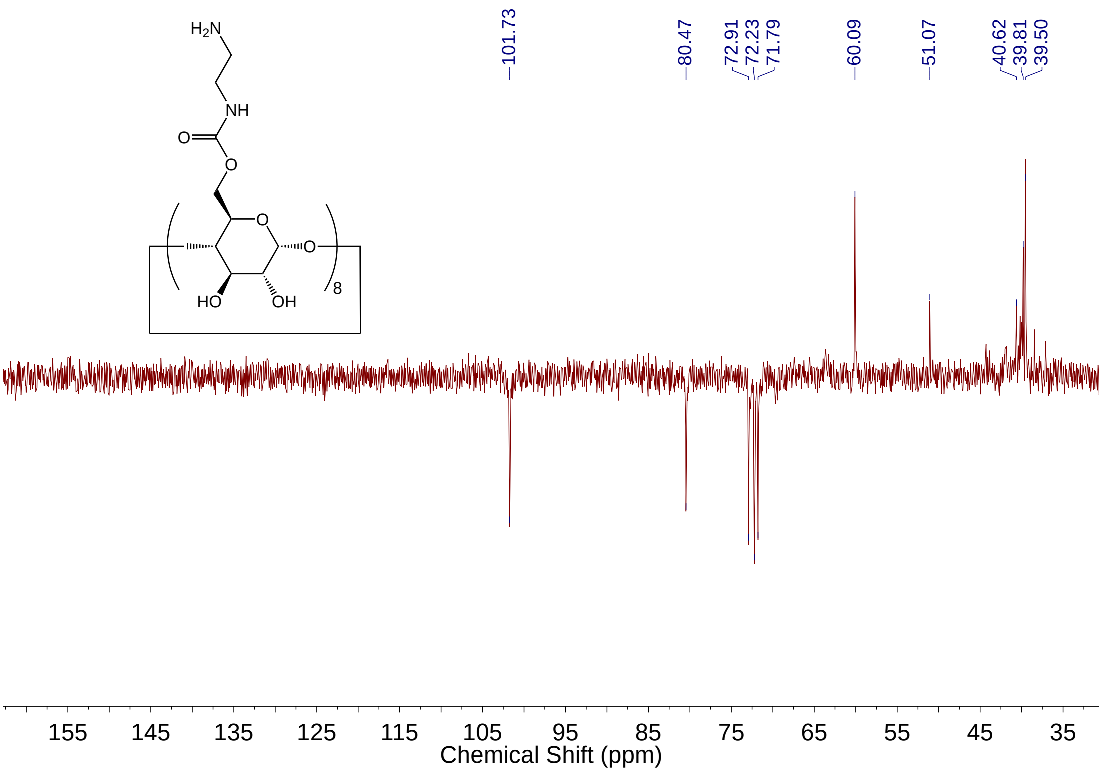


**Supplementary Figure 14 | $^{13}$C DEPT-135 NMR spectrum of *γ*-CDA (3) in $D_2O$.**

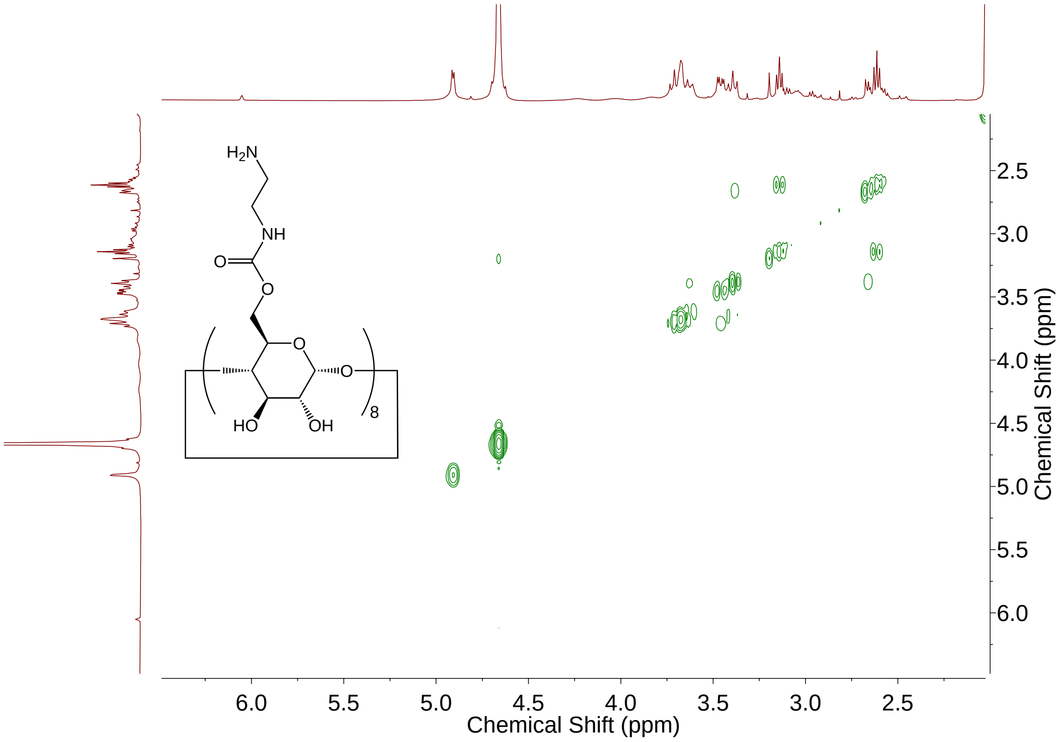


**Supplementary Figure 15 | 2D $^{1}$H-$^{1}$H COSY NMR spectrum of $\gamma$-CDA (3) in $D_2O$.**

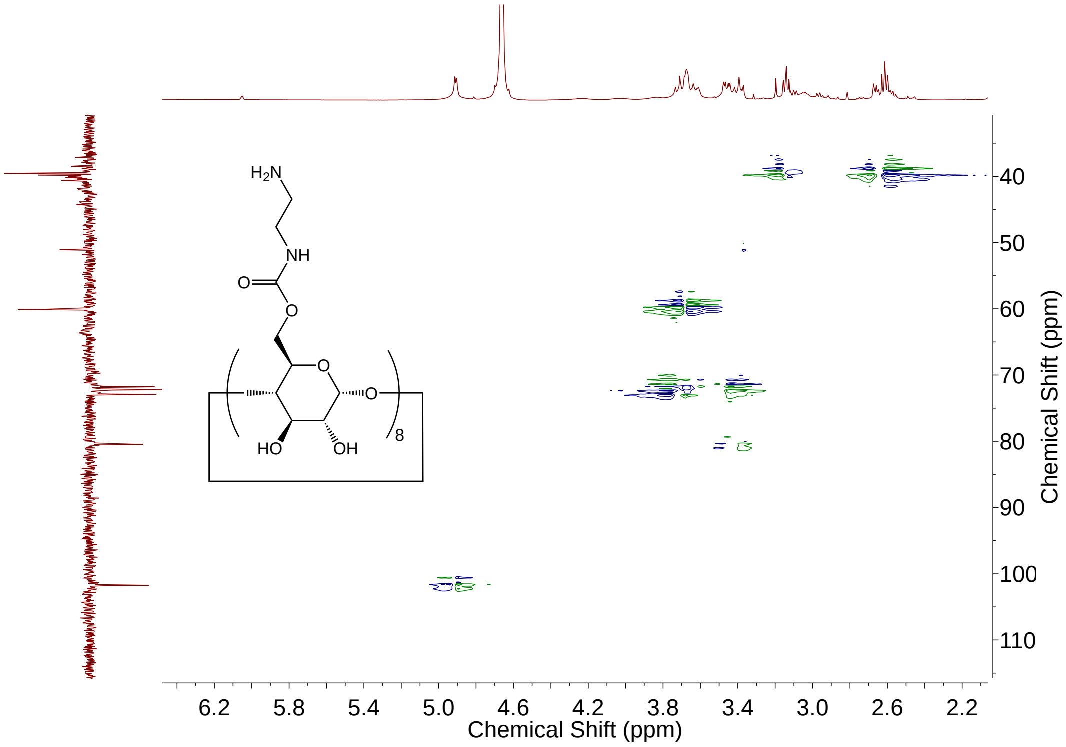


**Supplementary Figure 16 | 2D $^{1}$H-$^{13}$C HSQC NMR spectrum of $\gamma$-CDA (3) in $D_2O$.**

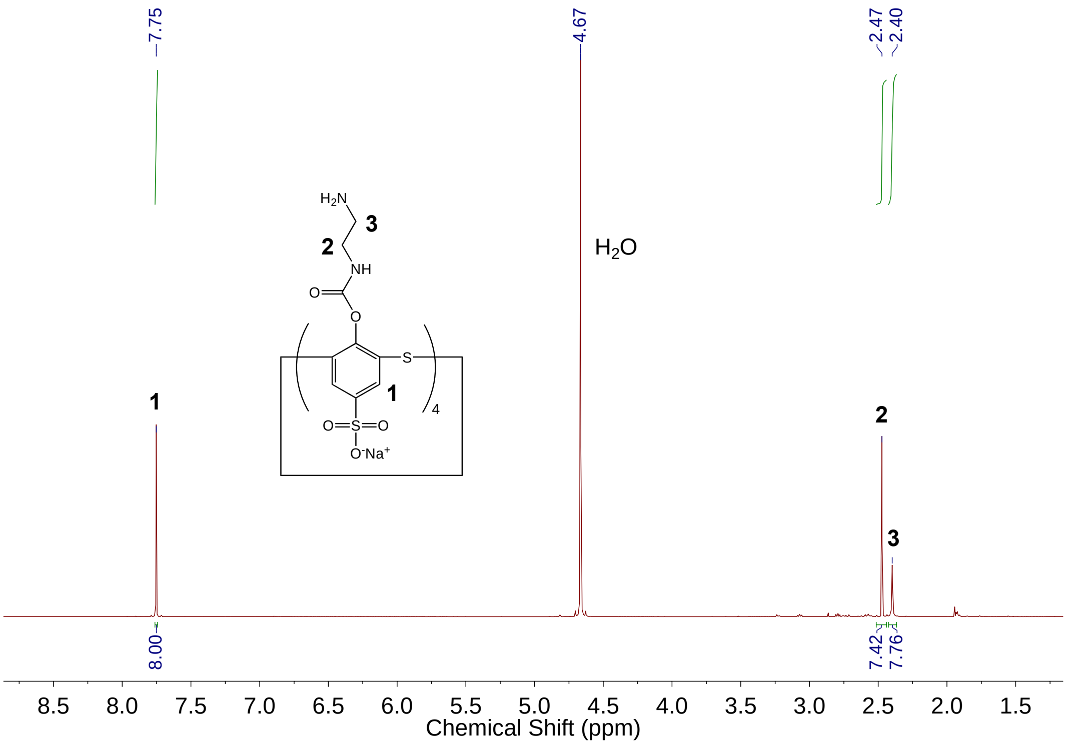


**Supplementary Figure 17 | $^{1}$H NMR spectrum of SC[4]AA (4) in $D_2O$.**

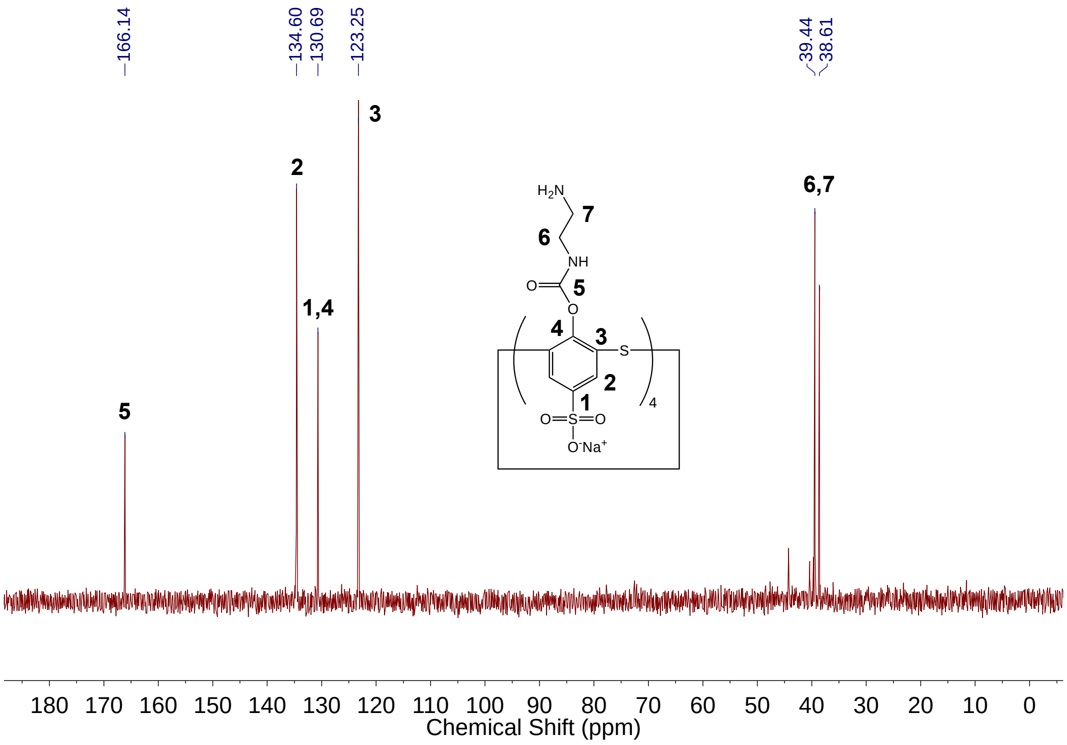


**Supplementary Figure 18 | $^{13}$C NMR spectrum of SC[4]AA (4) in $D_2O$.**

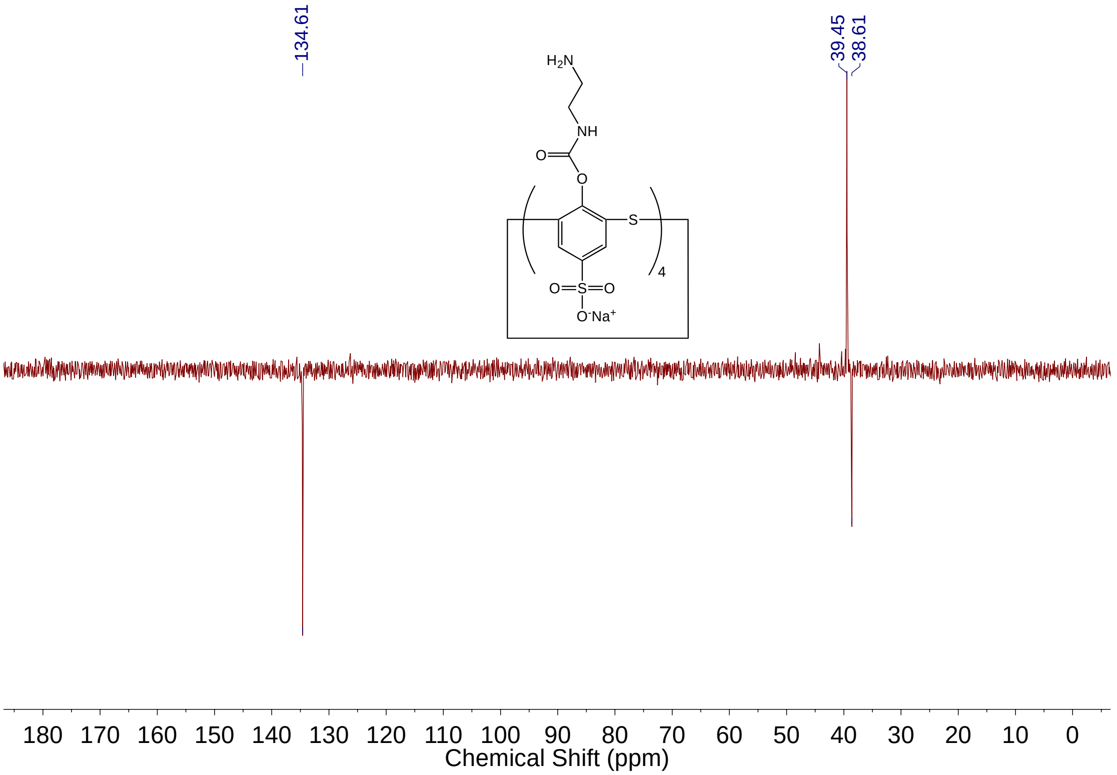


**Supplementary Figure 19 | $^{13}$C DEPT-135 NMR spectrum of SC[4]AA (4) in $D_2O$.**

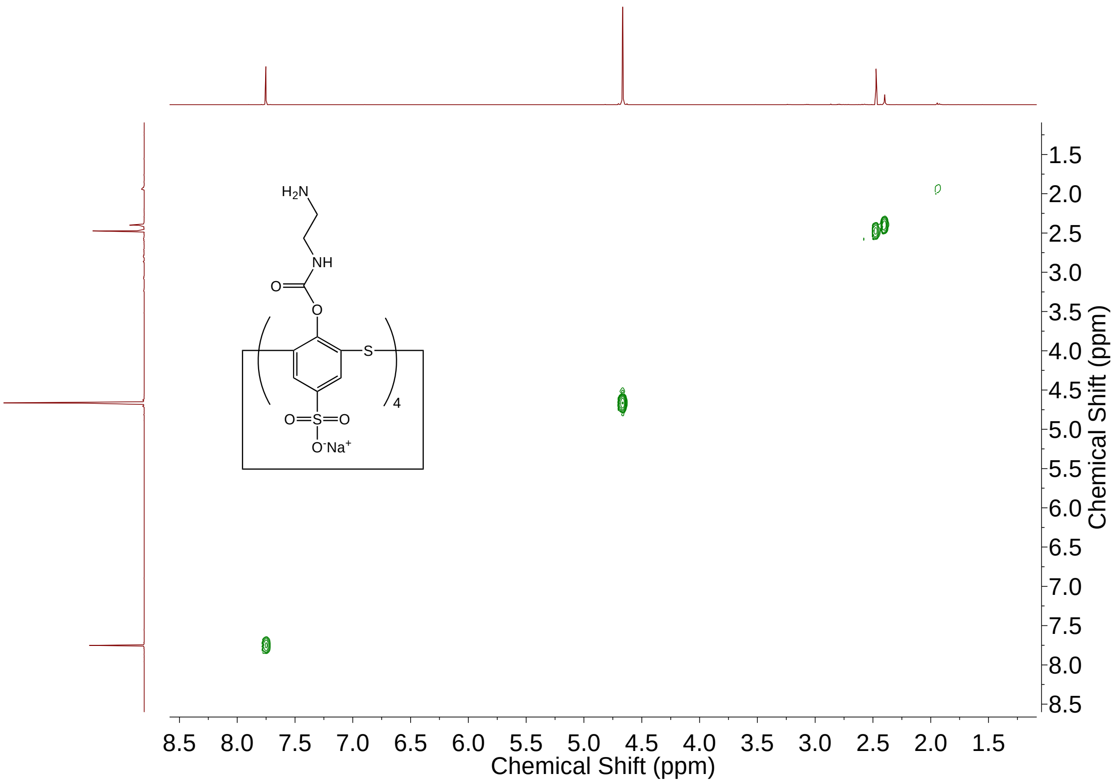


**Supplementary Figure 20 | 2D $^{1}$H-$^{1}$H COSY NMR spectrum of SC[4]AA (4) in $D_2O$.**

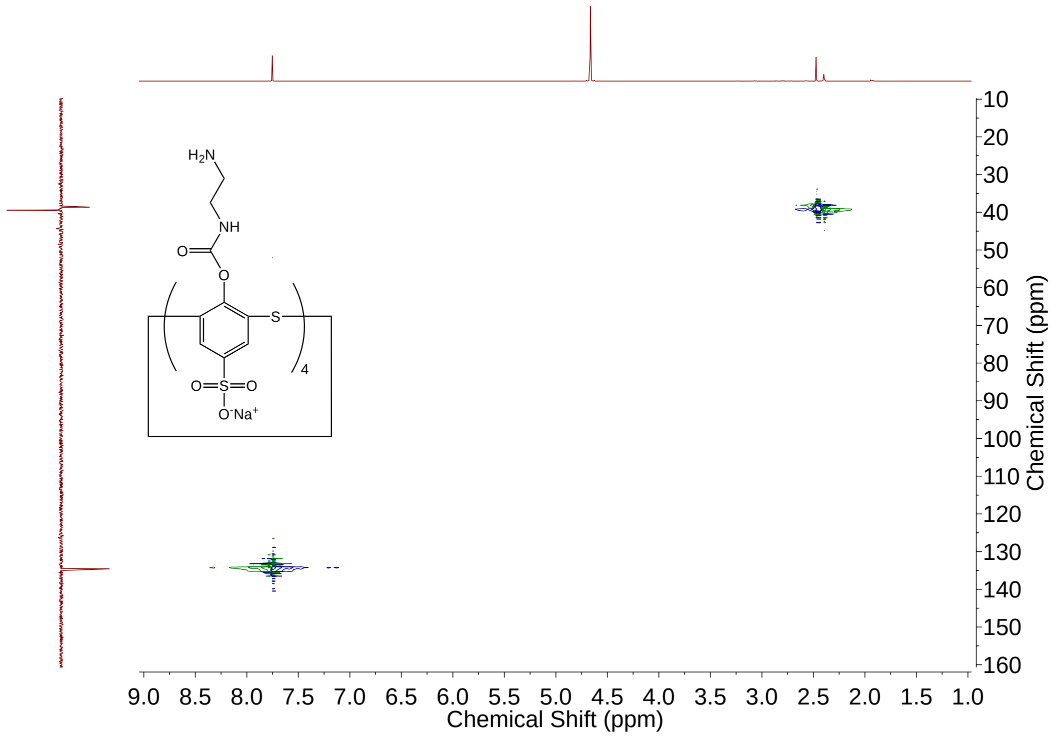


**Supplementary Figure 21 | 2D $^{1}$H-$^{13}$C HSQC NMR spectrum of SC[4]AA (4) in $D_2O$.**

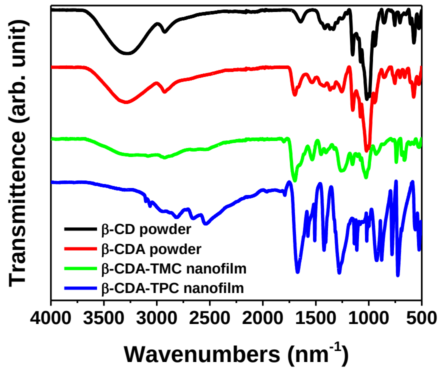


**Supplementary Figure 22 | Fourier-transform infrared (FT-IR) spectra of the precursors, amino derivatives, and the resulting nanofilms.** These include unfunctionalised *β*-cyclodextrin (*β*-CD) powder, synthesized amino-functionalised *β*-cyclodextrin (*β*-CDA) powder, and resulting nanofilms (*β*-CDA-TMC, and *β*-CDA-TPC) made from 0.1 wt.% *β*-CDA with 0.1 wt.% trimesoyl chloride (TMC) and 0.1 wt.% terephthaloyl chloride (TPC) respectively.

**Supplementary Table 1.** Functional groups analysed from FT-IR for unfunctionalised *β*-cyclodextrin (*β*-CD) powder, amino-functionalised *β*-cyclodextrin (*β*-CDA) powder, *β*-CDA-TMC nanofilm, and *β*-CDA-TPC nanofilm.

| Wavenumbers ($cm^{-1}$) –functional group | *β*-CD powder | *β*-CDA powder | *β*-CDA-TMC nanofilm | *β*-CDA-TPC nanofilm |
|---|---|---|---|---|
| 3290-(O-H) alcohol | √ | √ | √ | √ |
| 2930-(C-H) alkane | √ | √ | √ | √ |
| 1800-(C=O) carboxylic acid | × | × | √ | √ |
| 1700-(C=O) amide | × | √ | √ | √ |
| 1610-(C=C) conjugated alkene | × | × | √ | √ |
| 1530-(C-N) amide | × | √ | √ | √ |
| 1020-(C-O-C) ether | √ | √ | √ | √ |

√ indicates the presence of the functional group. × indicates the absence of the functional group.

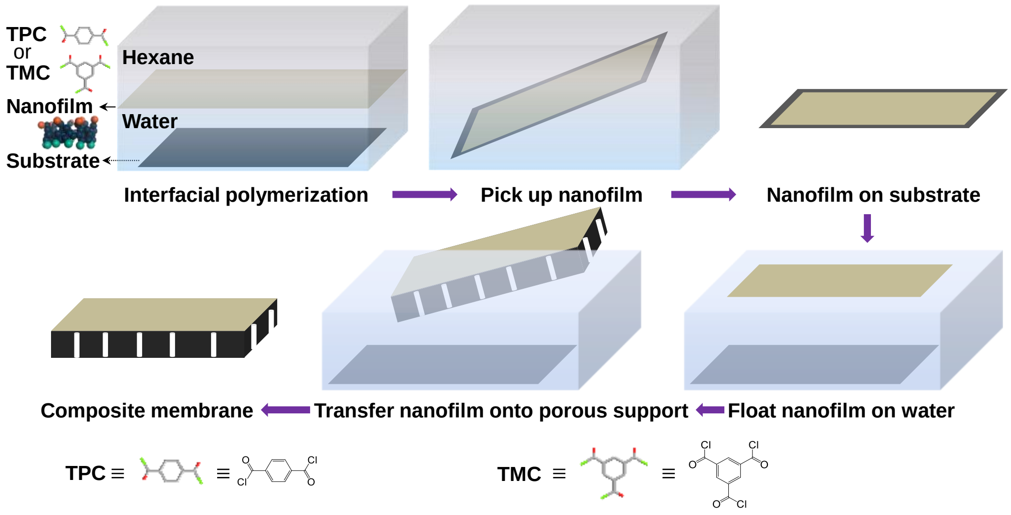


**Supplementary Figure 23 | Schematic of interfacial polymerisation at a free interface**. The reaction occurs between an aqueous phase containing amino-functionalised macrocycles and hexane phase containing terephthaloyl chloride (TPC) or trimesoyl chloride (TMC).

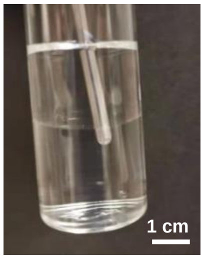


**Supplementary Figure 24 | Photograph of the free interface between an aqueous phase containing unfunctionalised *β*-cyclodextrin and a hexane phase containing terephthaloyl chloride (TPC).** No film was observed at the interface for this combination.

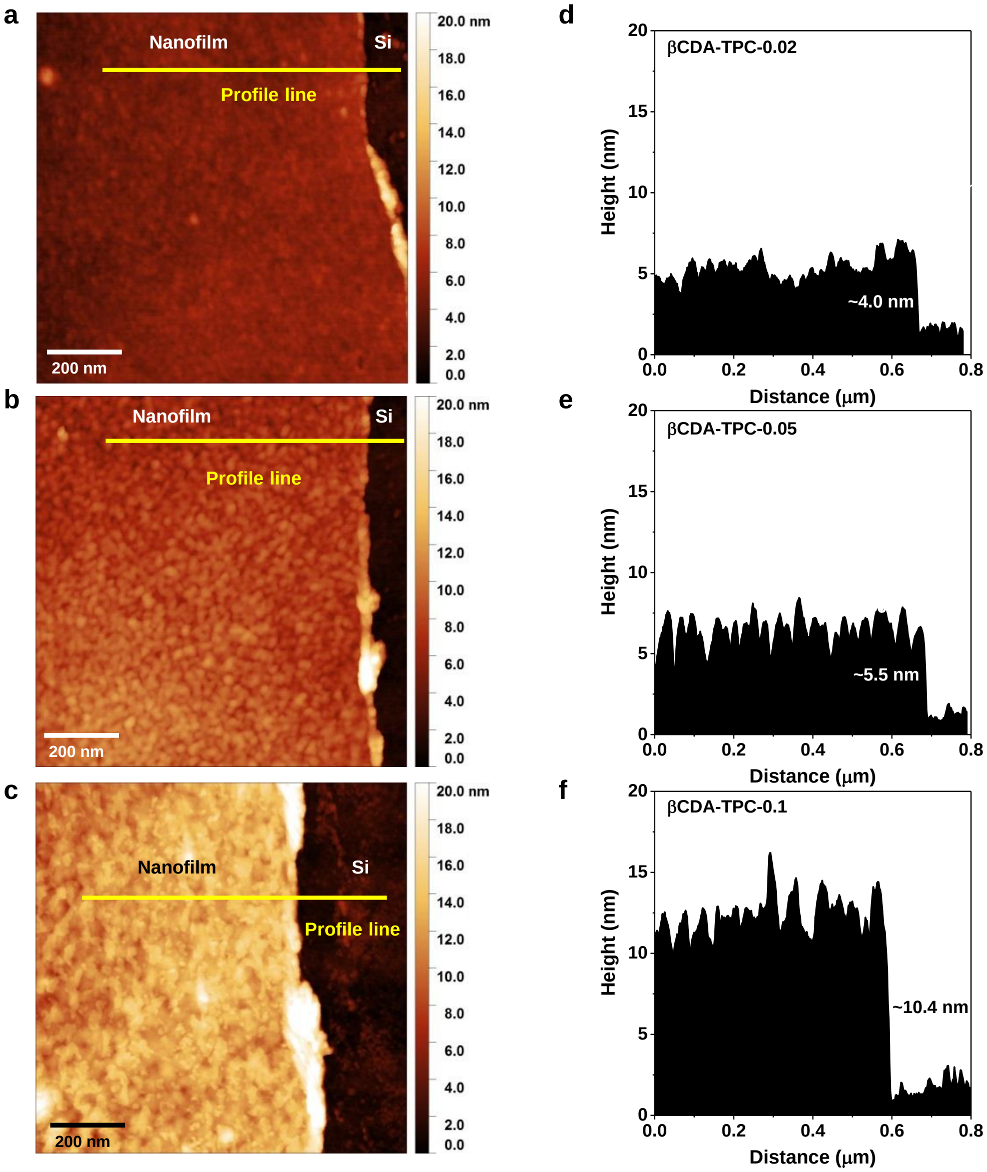


**Supplementary Figure 25 | Atomic force microscopy (AFM) height images of the nanofilms**. They were made from **a,** 0.02 wt.%, **b,** 0.05 wt.%, and **c,** 0.1 wt.% amino-functionalised *β*-cyclodextrin (*β*-CDA) and 0.1 wt.% terephthaloyl chloride (TPC) reacted for 1 min. **d-f,** Height along profile lines for respective nanofilms. The nanofilms were fabricated at the free interface and transferred onto silicon (Si) wafers.

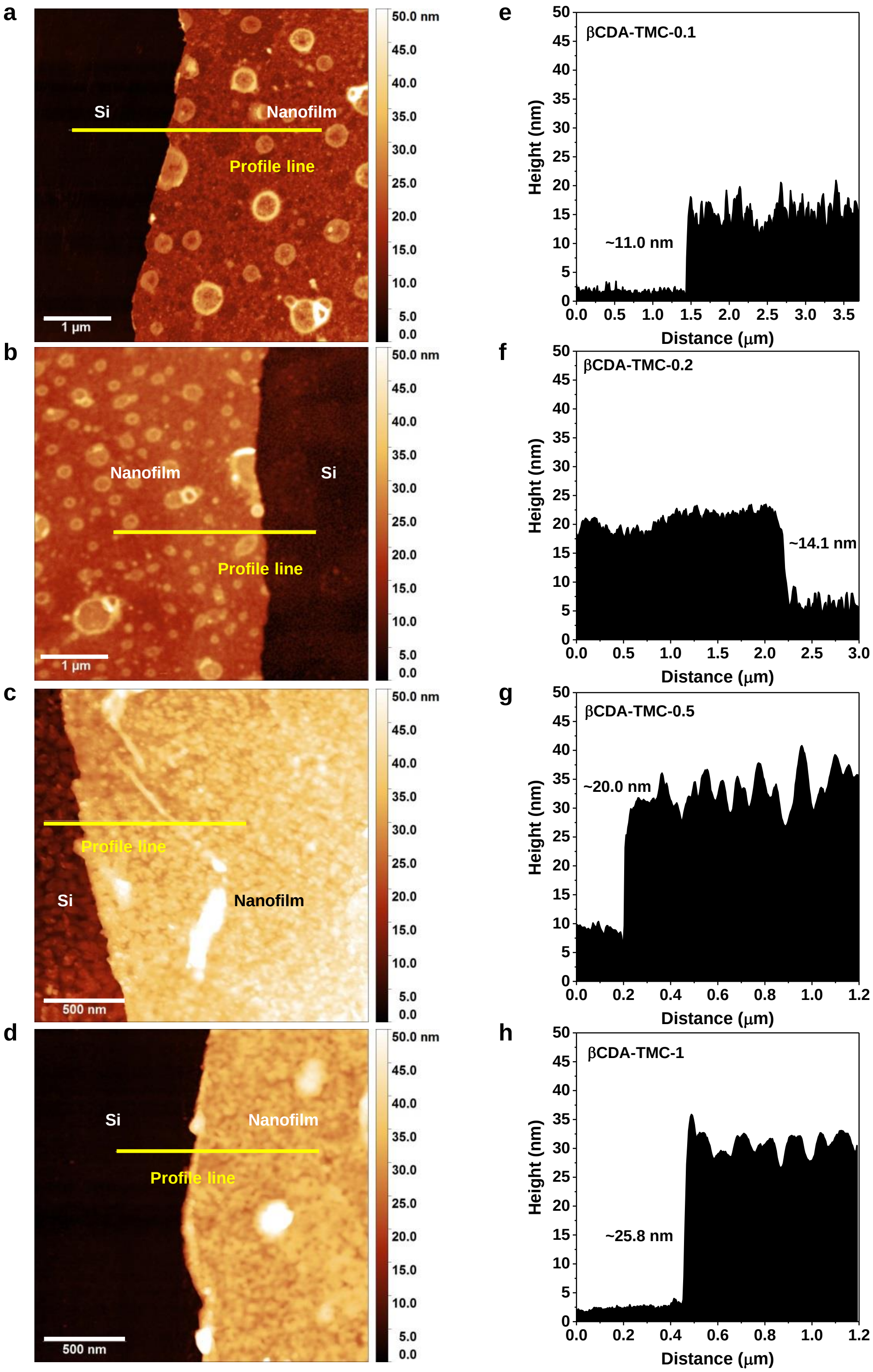


**Supplementary Figure 26 | AFM height image of the nanofilms.** They were made from **a,** 0.1 wt.%, **b,** 0.2 wt.%, **c,** 0.5 wt.%, and **d,** 1.0 wt.% amino-functionalised *β*-CDA and 0.1 wt.% trimesoyl chloride (TMC) reacted for 1 min. **e-h,** Height along profile lines scanned for respective nanofilms.

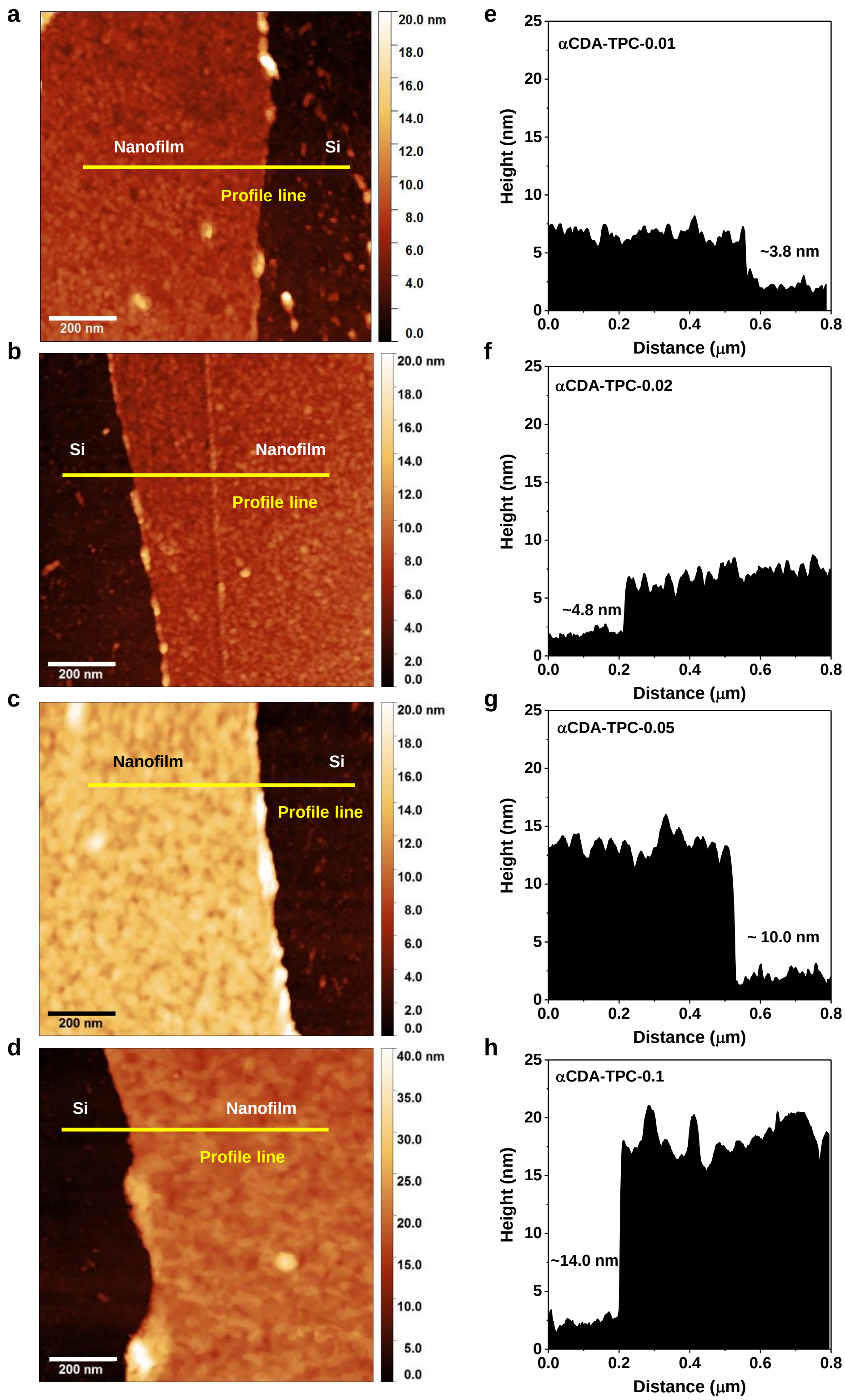


**Supplementary Figure 27 | AFM height image of the nanofilms**. They were made from **a,** 0.01 wt.%, **b,** 0.02 wt.%, **c,** 0.05 wt.%, **d,** 0.1 wt.% amino-functionalised *α*-cyclodextrin (*α*-CDA) and 0.1 wt.% TPC reacted for 1 min. **e-h,** Height along profile lines scanned for respective nanofilms.

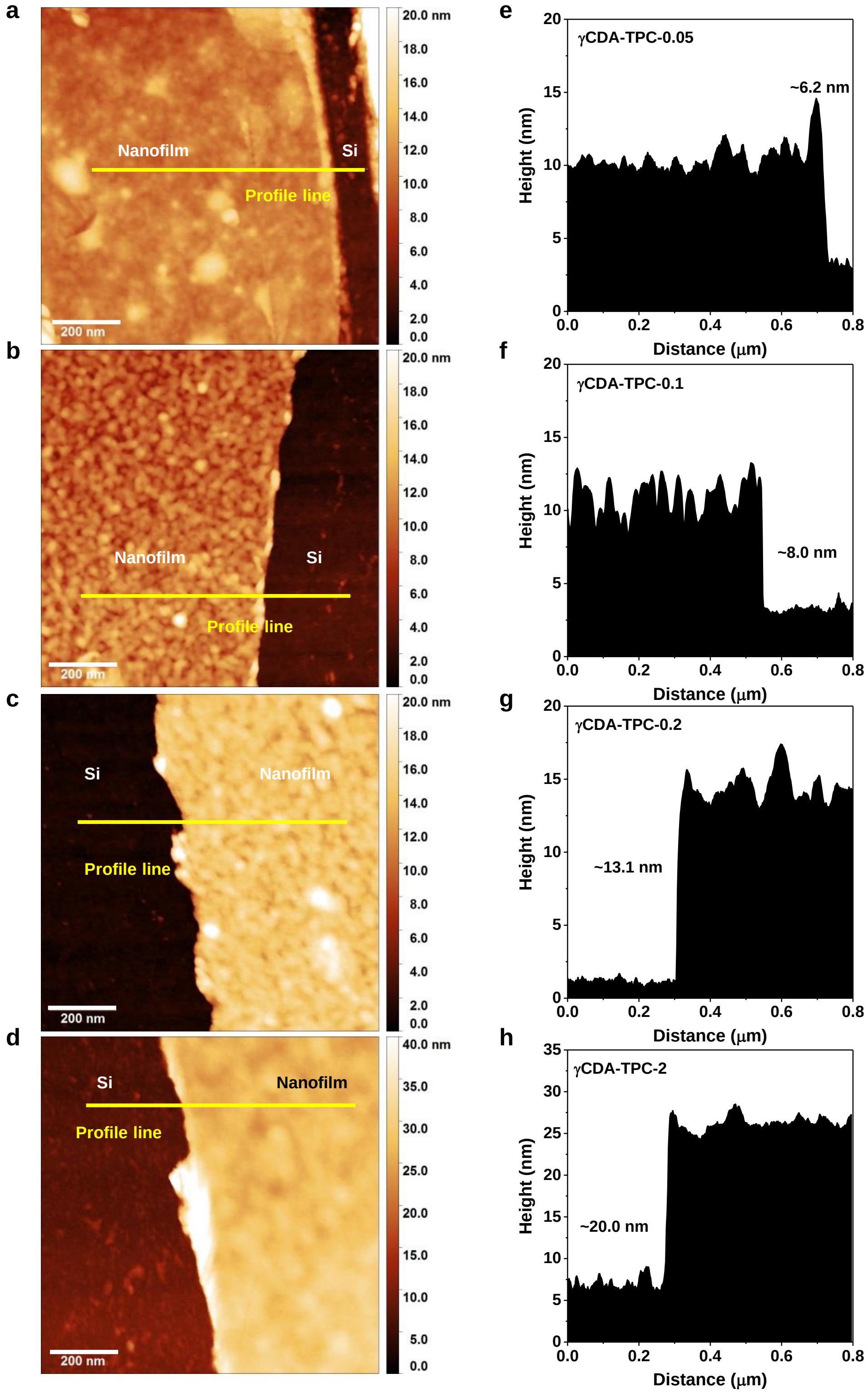


**Supplementary Figure 28 | AFM height image of the nanofilms.** They were made from **a,** 0.05 wt.%, **b,** 0.1 wt.%, **c,** 0.2 wt.%, and **d,** 2 wt.% amino-functionalised *γ*-cyclodextrin (*γ*-CDA) and 0.1 wt.% TPC reacted for 1 min. **e-h,** Height along profile lines scanned for respective nanofilms.

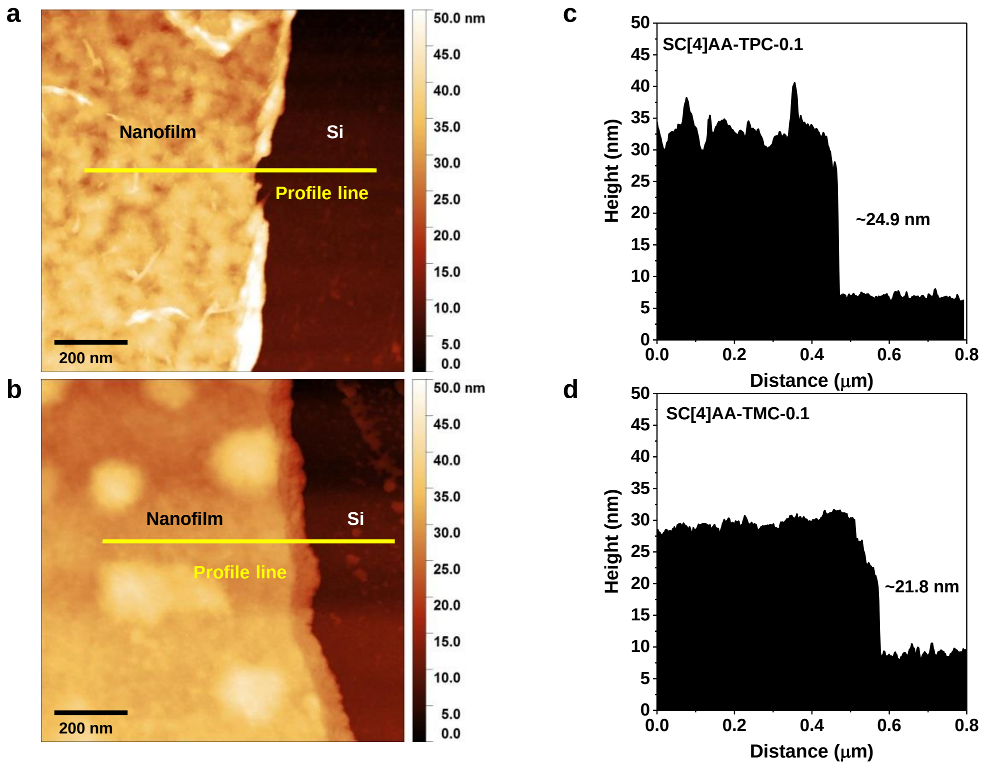


**Supplementary Figure 29 | AFM height image of the nanofilms.** They were made from 0.1 wt.% amino-functionalised 4-sulfocalix[4]arene (SC[4]AA) with **a,** 0.1 wt.% TPC and **b,** 0.1 wt.% TMC reacted for 20 min. **c** and **d,** Height along profile lines scanned for respective nanofilms. The nanofilm thicknesses were one order of magnitude thinner than those fabricated from unfunctionalised precursors[41].

**Supplementary Table 2.** Upper (narrow) and lower (wide) rim dimensions of macrocycles.

| **Property** | ***α*-CDA*** | ***β*-CDA*** | **γ-CDA*** | **SC[4]AA†** |
|---|---|---|---|---|
| Upper rim diameter (nm) | 0.45 | 0.61 | 0.77 | 0.57 |
| Lower rim diameter (nm) | 0.57 | 0.78 | 0.95 | 0.57 |

*Dimensions adapted from the reported literature[42]. †Dimensions from molecular simulation, please refer to Supplementary Figure 48.

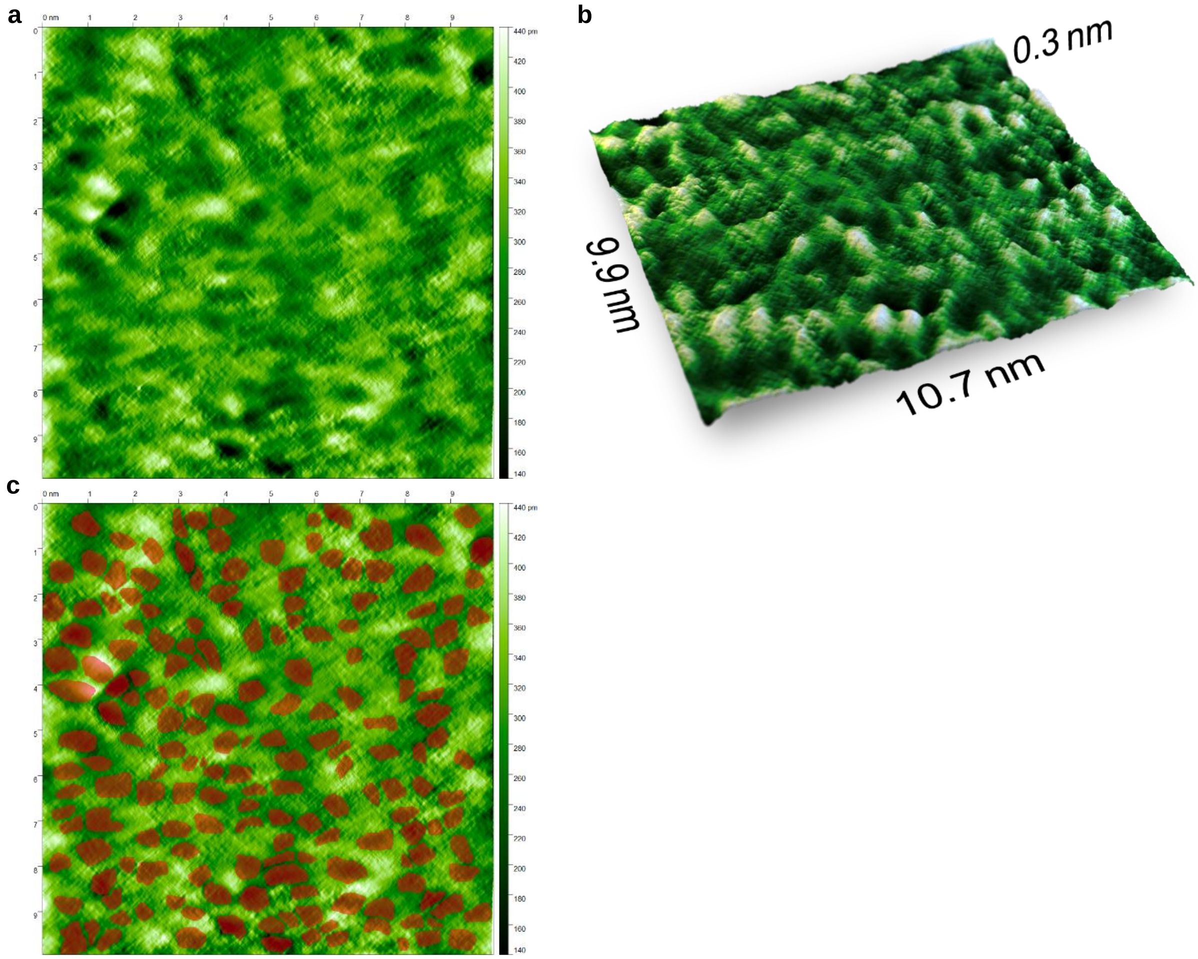


**Supplementary Figure 30 | UHV AFM height images of nanofilms made from medium cavity *β*-CDA.** Original **a,** 2D, **b,** 3D images, and **c,** 2D image with pores being marked.

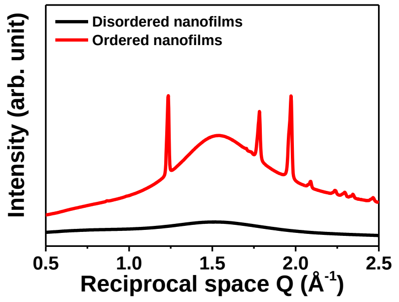


**Supplementary Figure 31 | Powder X-ray diffraction of the nanofilm powders.** They were made from disordered nanofilms (*β*-CDA-TMC-0.1) and ordered nanofilms (*β*-CDA-TPC-0.1).

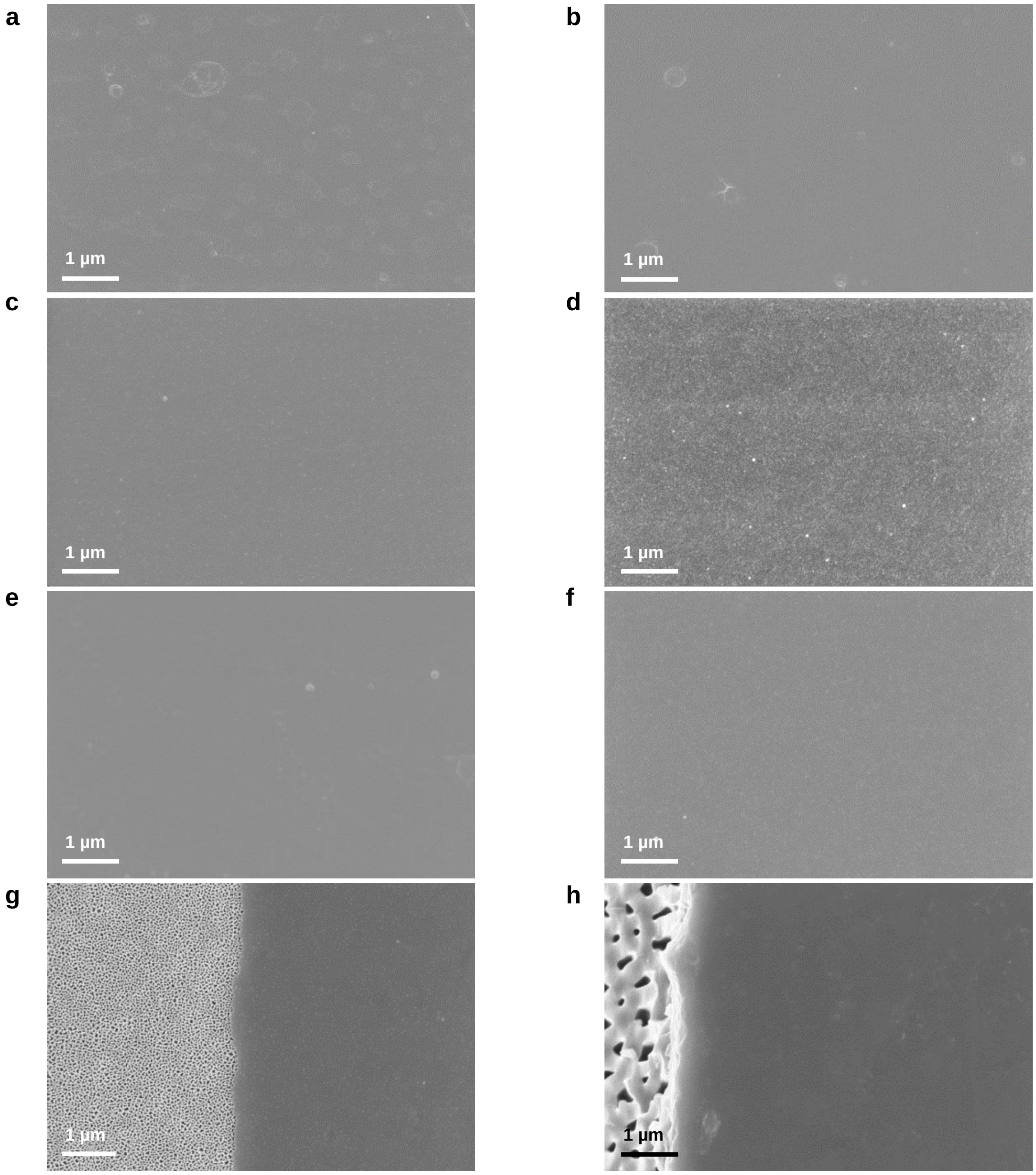


**Supplementary Figure 32 | SEM surface images of the nanofilms.** These include nanofilms (*β*-CDA-TMC-0.1) made from 0.1 wt.% amino-functionalised *β*-cyclodextrin (*β*-CDA) and 0.1 wt.% TMC revealing **a,** the front-surface-up and **b,** the back-surface-up transferred onto PAN supports. SEM images of the nanofilms (*β*-CDA-TPC-0.1) made from 0.1 wt.% *β*-CDA and 0.1 wt.% TPC revealing **c,** the front-surface-up and **d,** the back-surface-up transferred onto PAN supports. SEM images of **e,** the nanofilm (*β*-CDA-TMC-0.05) made from 0.05 wt.% *β*-CDA and 0.1 wt.% TMC and **f,** the nanofilm (*β*-CDA-TPC-0.05) made from 0.05 wt.% *β*-CDA and 0.1 wt.% TPC transferred onto PAN supports. SEM surface images of **g,** the nanofilm (*β*-CDA-TPC-0.1) made from 0.1 wt.% *β*-CDA and 0.1 wt.% TPC and **h,** the nanofilm (*α*-CDA-TPC-0.1) made from 0.1 wt.% *α*-CDA and 0.1 wt.% TPC transferred onto alumina supports.

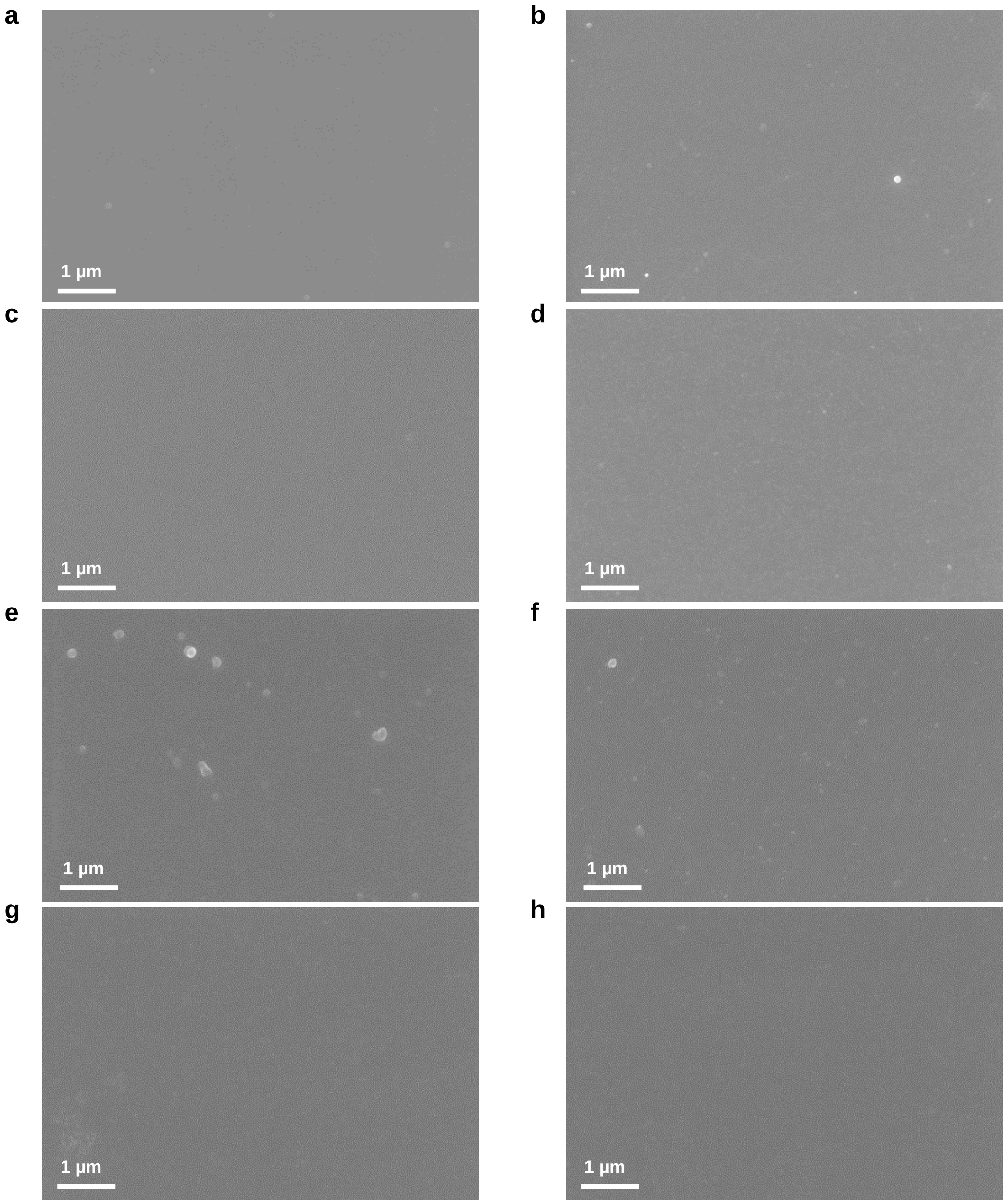


**Supplementary Figure 33 | SEM surface images of the nanofilms.** These include nanofilms made from **a,** 0.05 wt.% $\alpha$-CDA ($\alpha$-CDA-TPC-0.05), **b,** 0.1 wt.% $\alpha$-CDA ($\alpha$-CDA-TPC-0.1), **c,** 0.05 wt.% $\gamma$-CDA ($\gamma$-CDA-TPC-0.05), **d,** 0.1 wt.% $\gamma$-CDA ($\gamma$-CDA-TPC-0.1), **e,** 0.2 wt.% $\gamma$-CDA ($\gamma$-CDA-TPC-0.2), and **f,** 2 wt.% $\gamma$-CDA ($\gamma$-CDA-TPC-2) with 0.1 wt.% terephthaloyl chloride (TPC) reacted for 1 min and transferred onto PAN supports. SEM images of **g,** the nanofilms (SC[4]AA-TPC-0.1) made from 0.1 wt.% amino-functionalised 4-sulfocalix[4]arene (SC[4]AA) reacted with 0.1 wt.% TPC and **h,** the nanofilms (SC[4]AA-TMC-0.1) made from 0.1 wt.% SC[4]AA reacted with 0.1 wt.% TMC for 20 min and transferred onto PAN supports.

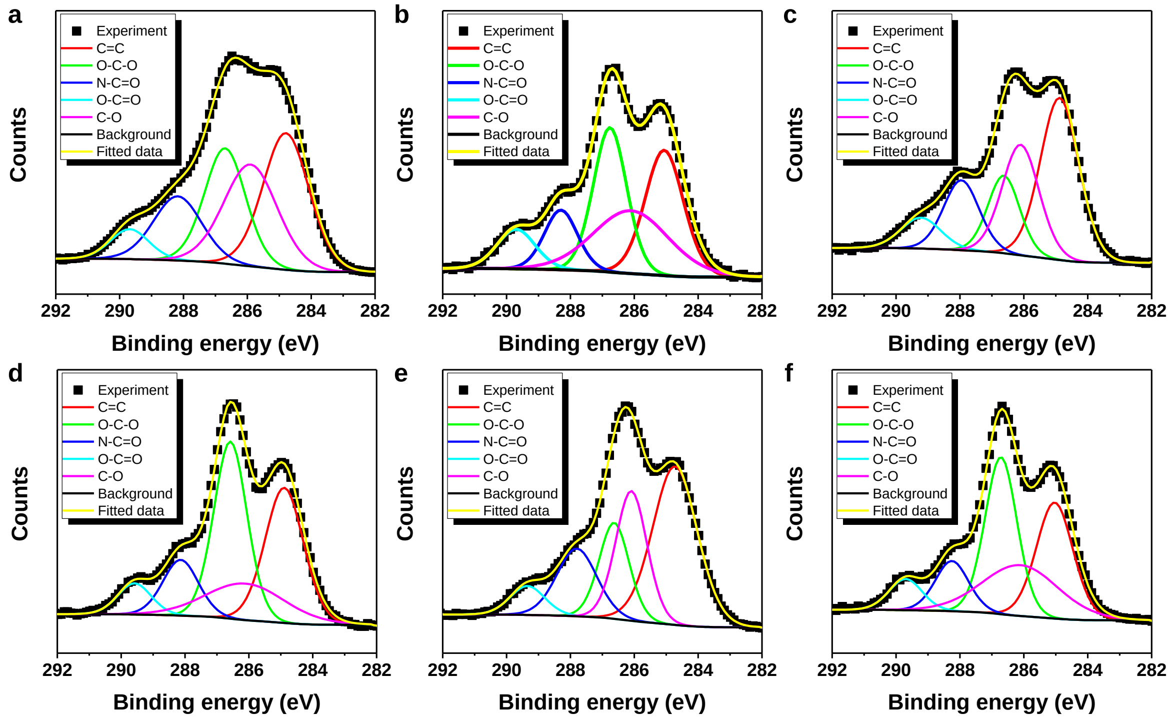


**Supplementary Figure 34 | X-ray photoelectron spectroscopy (XPS) C1s spectrum of the nanofilms.** They were made from **a,** 0.1 wt.% amino-functionalised *α*-cyclodextrin (*α*-CDA) and terephthaloyl chloride (TPC) (*α*-CDA-TPC-0.1), **b,** 0.1 wt.% *β*-CDA and TPC (*β*-CDA-TPC-0.1), **c,** 0.1 wt.% *β*-CDA and trimesoyl chloride (TMC) (*β*-CDA-TMC-0.1), **d,** 0.1 wt.% *γ*-CDA and TPC (*γ*-CDA-TPC-0.1), **e,** 0.2 wt.% *γ*-CDA and TPC (*γ*-CDA-TPC-0.2), and **f,** 2 wt.% *γ*-CDA and TPC (*γ*-CDA-TPC-2). Concentration of acyl chloride for all nanofilms were controlled at 0.1 wt.%.

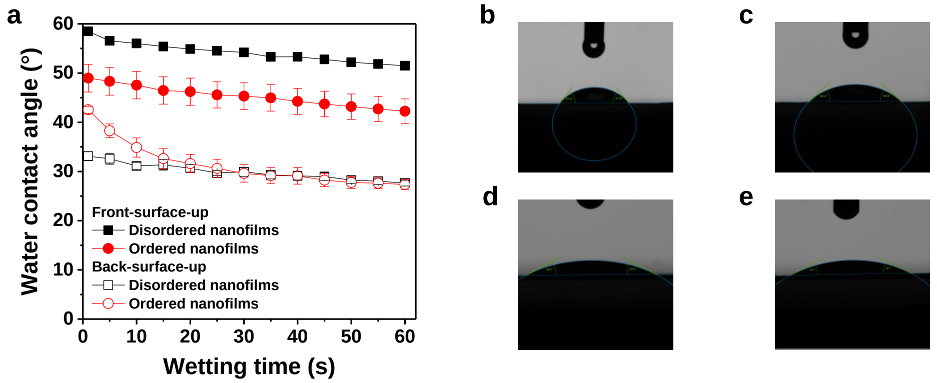


**Supplementary Figure 35 | Water contact angle of disordered nanofilms (*β*-CDA-TMC-0.1) and ordered nanofilms (*β*-CDA-TPC-0.1) with their front or back surfaces facing up. a,** Measurement of water contact angle over wetting time of 60 sec. Photographs of water droplets on the surface of **b,** disordered nanofilms and **c,** ordered nanofilms with front-surface-up, and **d,** disordered nanofilms and **e,** ordered nanofilms with back-surface-up. High water contact angle reveals a more hydrophobic surface that creates potential for fast transport of non-polar solvent.

**Supplementary Table 3.** Properties of dye molecules used for OSN. Charge 0 denotes neutral charge, - denotes negative charge, and + denotes positive charge.

| Dye molecule name | Structure | Dimension (nm × nm) | Molecular weight (g mol$^{-1}$) | Charge |
|---|---|---|---|---|
| 4-nitrophenol (4NP) | 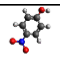 | 0.25 × 0.63 | 139 | 0 |
| Azobenzene (AZB) | 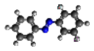 | 0.43 × 0.90 | 182 | 0 |
| Methyl orange (MO) | 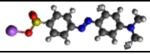 | 0.51 × 1.5 | 327 | - |
| Safranin O (SO) | 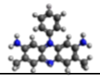 | 0.73 × 0.97 | 351 | + |
| Congo red (CR) | 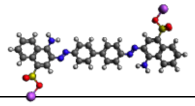 | 0.89 × 2.4 | 696 | - |
| Sunset yellow (SY) | 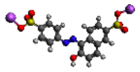 | 1.1 × 1.7 | 452 | - |
| Acid fuchsin (ACF) | 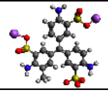 | 1.2 × 1.3 | 585 | - |
| Brilliant blue R (BB) | 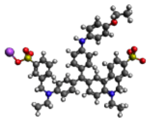 | 1.4 × 2.1 | 826 | - |

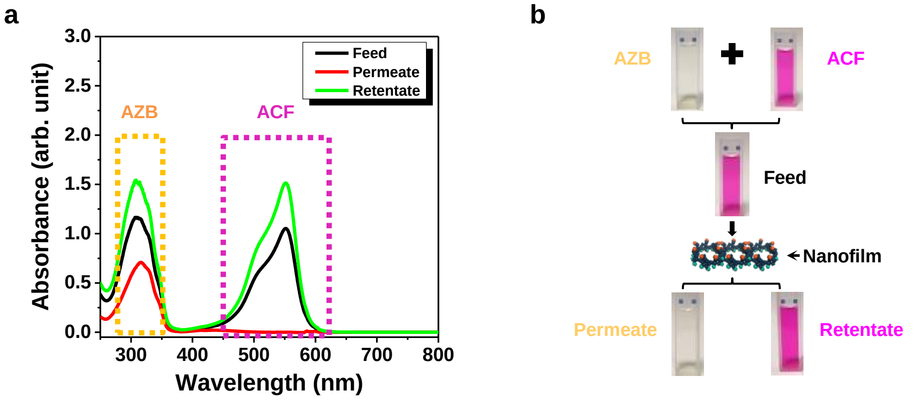


**Supplementary Figure 36 | Rejection of a feed mixture containing azobenzene (AZB) and acid fuchsin (ACF) in methanol through β-CDA-TPC-0.1 membrane. a,** UV-vis spectra and **b,** photos of the feed, permeate, and retentate.

**Supplementary Table 4**. Solubility parameter and viscosity of various solvents.

| Solvent | Solubility parameter, $\delta$, (MPa$^{0.5}$)* | Viscosity, $\mu$, ($10^{-3}$ Pa.s)* |
|---|---|---|
| Water | 48.0 | 0.89 |
| Acetonitrile | 24.6 | 0.32 |
| Methanol | 29.7 | 0.49 |
| Acetone | 20.1 | 0.316 |
| Hexane | 14.4 | 0.297 |
| Heptane | 15.3 | 0.33 |
| Toluene | 18.2 | 0.52 |

*The values were adapted from the literature[2,43].

**Supplementary Table 5.** Performance of commercial polyamide-based nanofiltration membranes and composite membranes comprising nanofilms made from amino-functionalised macrocycles and acyl chloride at a free interface.

| **Membrane (macrocycle-acyl chloride-wt.%-remark)** | **Pure methanol permeance ($L\ m^{-2}\ h^{-1}\ bar^{-1}$)** | **Dye rejection (%)** | | | | | | | |
|---|---|---|---|---|---|---|---|---|---|
| | | **4NP (0.25 nm)** | **AZB (0.43 nm)** | **MO (0.51 nm)** | **SO (0.73 nm)** | **CR (0.89 nm)** | **SY (1.1 nm)** | **ACF (1.2 nm)** | **BB (1.4 nm)** |
| *α*-CDA-TPC-0.05 | 7.64 ± 0.1 | 18.0 ± 1.0 | 33.9 ± 0.9 | 93.0 ± 1.2 | 94.0 ± 0.9 | 98.6 ± 0.3 | 97.2 ± 0.6 | 99.4 ± 0.2 | 99.3 ± 0.3 |
| *α*-CDA-TPC-0.1 | 5.75 ± 0.2 | 8.4 ± 2.5 | 36.2 ± 4.9 | 90.3 ± 0.5 | 95.8 ± 0.8 | 99.3 ± 0.1 | 99.0 ± 0.1 | 99.5 ± 0.4 | 99.3 ± 0.3 |
| *α*-CDA-TPC-0.1-Al* | 9.51 ± 0.3 | 9.8 ± 1.5 | 18.3 ± 3.4 | 91.3 ± 1.2 | 96.3 ± 2.0 | 99.6 ± 0.2 | 96.0 ± 1.2 | 97.3 ± 1.4 | 99.7 ± 0.1 |
| *β*-CDA-TPC-0.05 | 9.84 ± 0.2 | 4.7 ± 0.7 | 28.3 ± 2.3 | 81.3 ± 5.2 | 95.5 ± 1.2 | 98.3 ± 0.3 | 98.3 ± 0.5 | 99.8 ± 0.1 | 99.6 ± 0.1 |
| *β*-CDA-TPC-0.1 | 6.26 ± 0.4 | 10.6 ± 0.3 | 27.7 ± 0.3 | 75.1 ± 3.9 | 96.2 ± 0.8 | 98.6 ± 0.4 | 96.0 ± 0.3 | 99.4 ± 0.3 | 99.8 ± 0.1 |
| *β*-CDA-TPC-0.1-back† | 10.4 ± 0.8 | 9.2 ± 1.7 | 23.8 ± 2.5 | 72.0 ± 4.0 | 90.7 ± 1.3 | 98.8 ± 0.5 | 94.4 ± 2.3 | 99.0 ± 0.3 | 98.5 ± 0.5 |
| *β*-CDA-TPC-0.1-Al* | 11.9 ± 0.4 | 15.6 ± 0.8 | 26.4 ± 1.6 | 83.2 ± 5.3 | 96.5 ± 2.1 | 98.3 ± 1.2 | 96.0 ± 1.7 | 99.7 ± 0.1 | 99.5 ± 0.1 |
| *β*-CDA-TPC-2-direct⁑ | 1.14 ± 0.1 | 42.1 ± 2.7 | 63.6 ± 3.1 | 92.6 ± 2.4 | 94.7 ± 3.3 | 99.0 ± 0.3 | 98.5 ± 0.7 | 98.0 ± 0.8 | 99.1 ± 0.2 |
| *β*-CDA-TMC-0.05 | 3.91 ± 0.1 | 6.5 ± 0.5 | 26.9 ± 2.1 | 92.4 ± 1.6 | 92.2 ± 1.2 | 98.4 ± 0.3 | 95.8 ± 0.9 | 98.2 ± 0.5 | 98.5 ± 0.6 |
| *β*-CDA-TMC-0.1 | 3.00 ± 0.5 | 10.0 ± 0.7 | 16.5 ± 2.1 | 93.0 ± 1.5 | 98.0 ± 0.4 | 99.8 ± 0.1 | 97.2 ± 0.7 | 99.2 ± 0.2 | 99.7 ± 0.1 |
| *β*-CDA-TMC-0.1-back† | 6.27 ± 0.3 | 23.2 ± 3.2 | 44.5 ± 2.0 | 93.6 ± 1.3 | 93.4 ± 1.6 | 98.9 ± 0.3 | 98.0 ± 0.4 | 99.0 ± 0.1 | 99.5 ± 0.1 |
| γ-CDA-TPC-0.05 | 9.87 ± 0.2 | 3.6 ± 1.1 | 12.3 ± 0.2 | 57.0 ± 5.3 | 58.2 ± 6.1 | 99.0 ± 0.1 | 91.1 ± 0.8 | 98.0 ± 0.3 | 99.2 ± 0.2 |
| γ-CDA-TPC-0.1 | 9.30 ± 0.5 | 7.4 ± 0.8 | 23.5 ± 4.4 | 60.0 ± 3.6 | 75.7 ± 2.1 | 98.6 ± 1.0 | 94.5 ± 1.6 | 99.7 ± 0.1 | 99.8 ± 0.1 |
| γ-CDA-TPC-0.2 | 1.92 ± 0.3 | 15.2 ± 1.0 | 58.9 ± 0.8 | 91.2 ± 3.7 | 94.6 ± 4.6 | 99.1 ± 0.2 | 96.7 ± 1.8 | 99 ± 0.1 | 99.8 ± 0.1 |
| γ-CDA-TPC-2 | 1.61 ± 0.2 | 40.5 ± 2.0 | 70.2 ± 3.5 | 96.7 ± 1.2 | 98.6 ± 1.0 | 99.0 ± 0.1 | 99.7 ± 0.1 | 99.7 ± 0.2 | 99.9 ± 0.1 |
| SC[4]AA-TPC-0.1‡ | 3.08 ± 0.4 | 25.3 ± 1.0 | 48.2 ± 2.3 | 84.3 ± 2.7 | 92.6 ± 0.8 | 94.3 ± 1.0 | 92.9 ± 0.9 | 94.2 ± 2.1 | 98.1 ± 0.7 |
| SC[4]AA-TMC-0.1‡ | 2.51 ± 0.2 | 22.7 ± 0.8 | 52.6 ± 1.9 | 95.7 ± 1.3 | 98.6 ± 0.4 | 99.6 ± 0.2 | 99.3 ± 0.1 | 99.6 ± 0.1 | 99.8 ± 0.1 |
| TriSep™ TS40⁂ | 8.65 ± 3.0 | 41.2 ± 1.7 | 48.0 ± 1.4 | 85.4 ± 4.9 | 48.7 ± 1.8 | 83.5 ± 13.5 | 90.9 ± 8.2 | 42.7 ± 12.8 | 45.1 ± 16.6 |
| Suez™ (GE) DK⁂ | 4.96 ± 0.5 | 38.2 ± 0.6 | 49.4 ± 6.4 | 93.3 ± 0.6 | 88.6 ± 1.2 | 98.9 ± 0.6 | 97.6 ± 0.5 | 75.8 ± 8.4 | 60.3 ± 29.6 |
| Synder Filtration™ NDX⁂ | 2.55 ± 0.3 | 49.4 ± 3.4 | 71.9 ± 5.0 | 96.3 ± 2.4 | 97.6 ± 0.7 | 98.6 ± 1.4 | 97.8 ± 2.1 | 95.7 ± 1.7 | 97.5 ± 0.5 |

Unless specified otherwise, nanofilms were made from amino-functionalised macrocycles and acyl chloride via interfacial polymerisation at a free interface reacted for 1 min and then transferred with the front surface facing up onto PAN supports to form composite membranes. *Nanofilms were transferred onto alumina supports to create composite membranes. †Nanofilms were flipped over and transferred with the back surface facing up (back-surface-up) onto the supports. ⁑Nanofilms were directly made on PAN supports via conventional interfacial polymerization. ‡Nanofilms were fabricated via interfacial reaction for 20 min. ⁂Commercial polyamide-based nanofiltration membranes. All experiments were carried out in a dead-end filtration cell at a constant pressure 10 bar and a constant temperature 25 °C under constant stirring at 250 rpm. Dye concentrations were maintained at 20 mg $L^{-1}$ throughout all experiments.

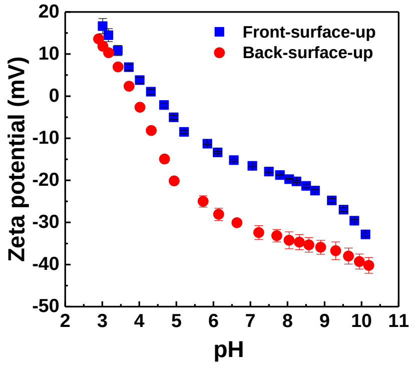


**Supplementary Figure 37 | Zeta potential of the front and back surfaces of the *β*-CDA-TMC-0.1 nanofilm.**

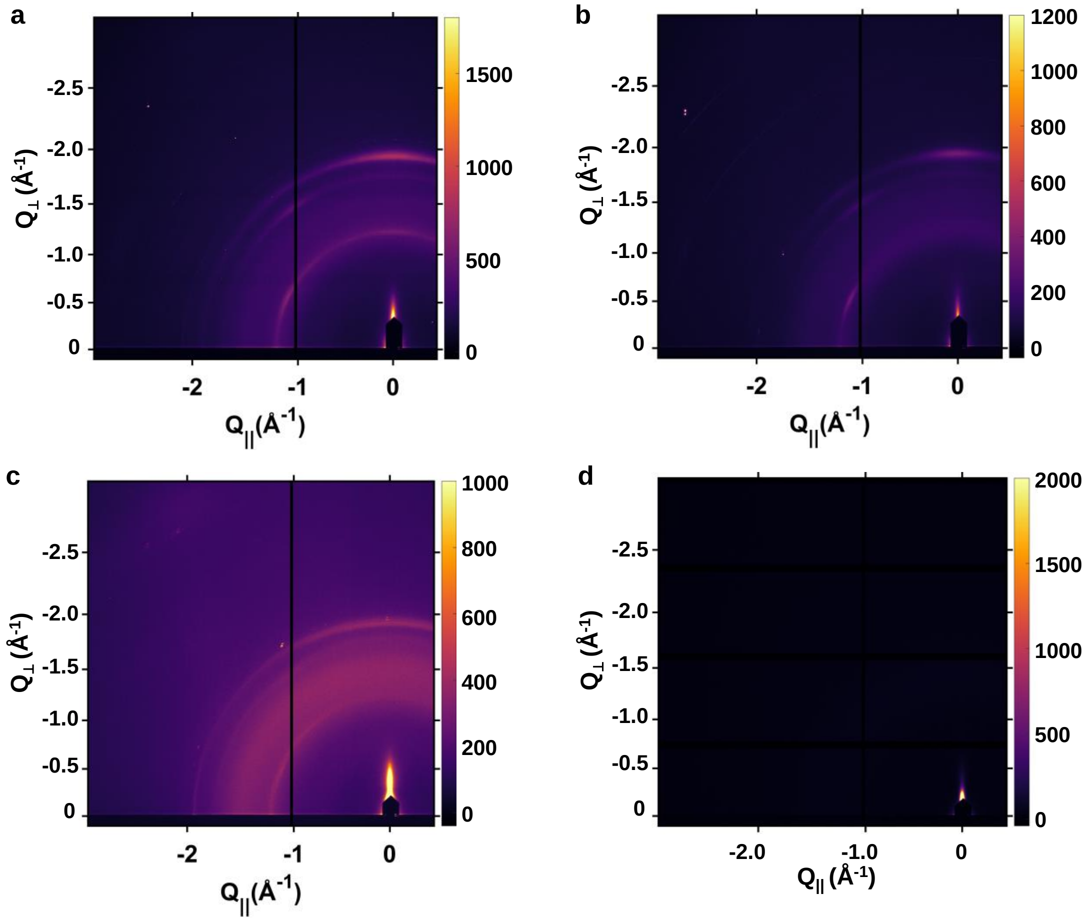


**Supplementary Figure 38 | GI-WAXS images of the nanofilms.** Two-dimensional images of nanofilms made from increasing *γ*-CDA concentrations **a,** 0.05 wt.% *γ*-CDA (*γ*-CDA-TPC-0.05), **b,** 0.1 wt.% *γ*-CDA (*γ*CDA-TPC-0.1), **c,** 0.2 wt.% *γ*-CDA (*γ*-CDA-TPC-0.2), and **d,** 2 wt.% *γ*-CDA (*γ*-CDA-TPC-2) with 0.1 wt.% terephthaloyl chloride (TPC). The corresponding one-dimensional image is shown in Fig. 3e in the main text.

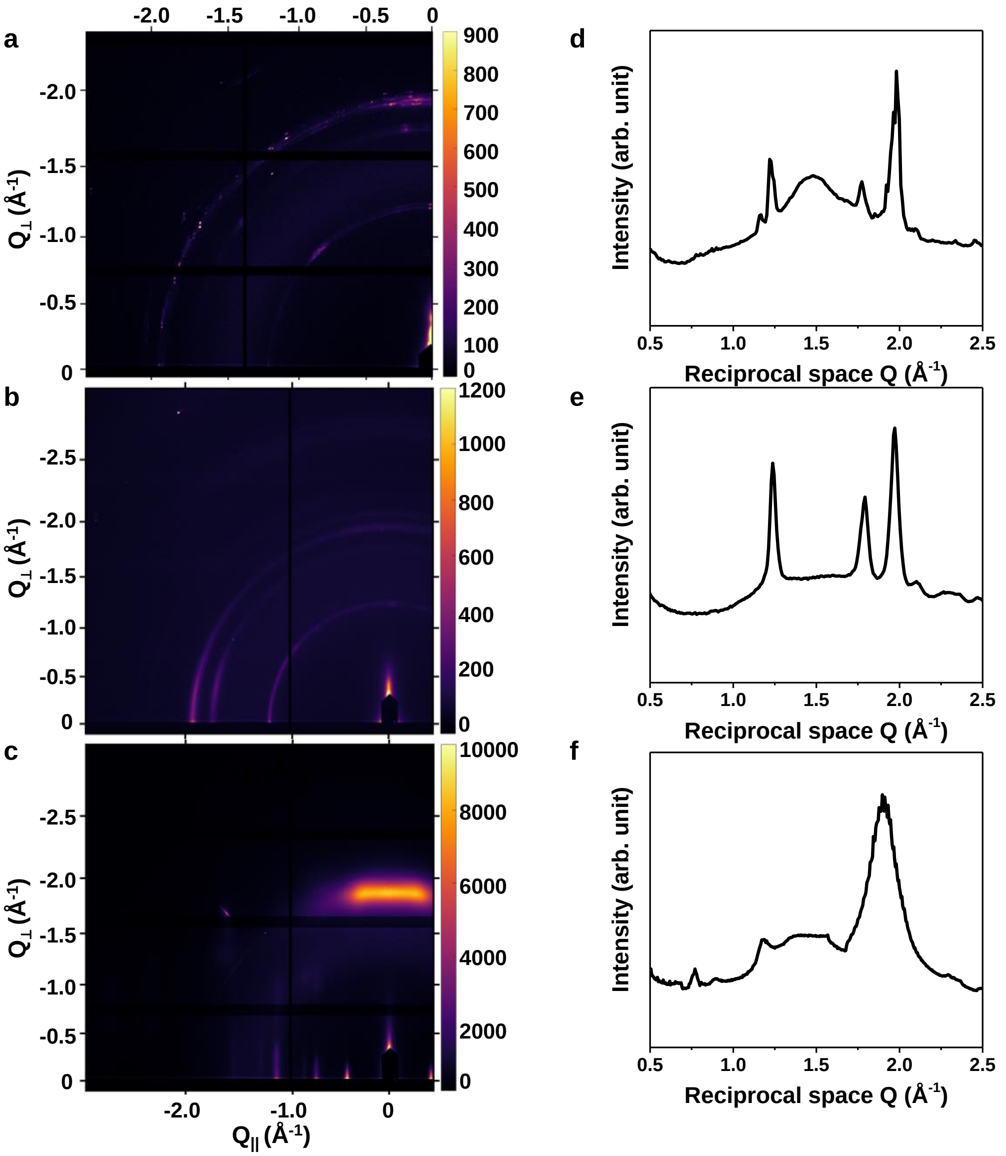


**Supplementary Figure 39 | GI-WAXS images of the nanofilms.** Two dimensional images of nanofilms made **a,** 0.01 wt.% *α*-CDA (*α*-CDA-TPC-0.01) and **b,** 0.01 wt.% *γ*-CDA (*γ*-CDA-TPC-0.01) with 0.1 wt.% TPC, and **c,** 0.01 wt.% *β*-CDA with 0.1 wt.% TMC (*β*-CDA-TMC-0.01). **d-f,** GI-WAXS one-dimensional images of the nanofilms in a-c respectively.

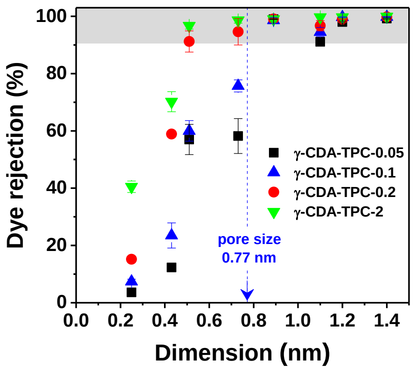


**Supplementary Figure 40 | Dye rejections of the composite membranes.** They comprise nanofilms made from various concentrations of $\gamma$-CDA with constant 0.1 wt.% TPC.

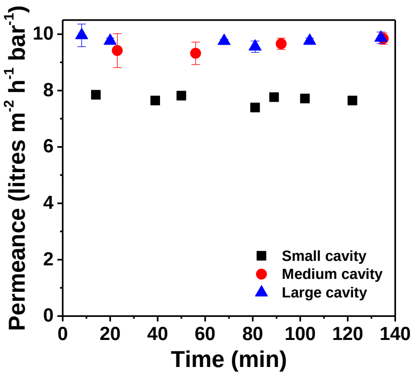


**Supplementary Figure 41 | Pure methanol permeance of the composite membranes.** They comprise nanofilms made from small cavity ($\alpha$-CDA-TPC), medium cavity ($\beta$-CDA-TPC), and large cavity ($\gamma$-CDA-TPC) at constant concentrations of 0.05 wt.% CDA and 0.1 wt.% TPC reacted for 1 min, and transferred onto PAN supports.

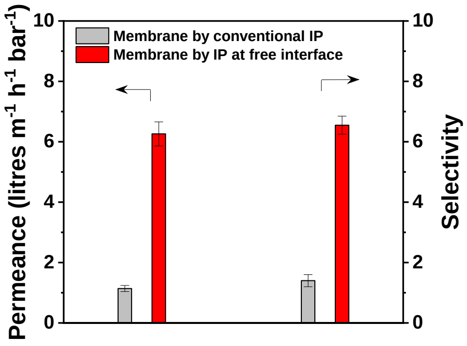


**Supplementary Figure 42 | Comparison of performance for composite membranes.** They comprise nanofilms made by conventional interfacial polymerisation (IP) directly on supports and by IP at free interface ($\beta$-CDA-TPC-0.1) and subsequently transferred onto supports. Selectivity of solute size 0.51 nm / 0.73 nm is plotted.

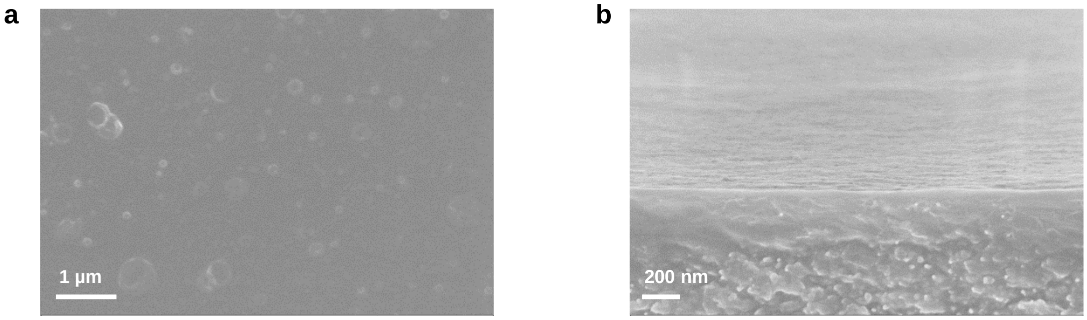


**Supplementary Figure 43 | SEM images of composite membranes fabricated directly on supports by conventional interfacial polymerisation. a,** surface, and **b,** cross sectional images.

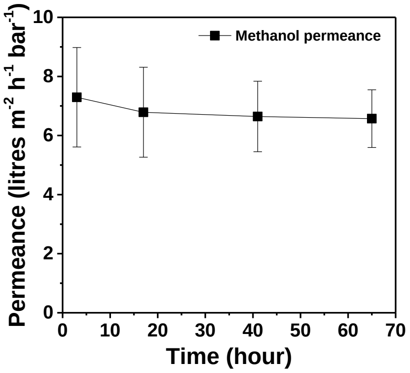


**Supplementary Figure 44 | Long-term methanol permeance for the composite membranes.** Membranes comprise nanofilms ($\beta$-CDA-TPC-0.1) made from 0.1 wt.% $\beta$-CDA and 0.1 wt.% TPC and then transferred onto PAN supports. The membranes were tested in a crossflow rig for 3 days at 25 °C under 10 bar with flowrate of 50 L $h^{-1}$.

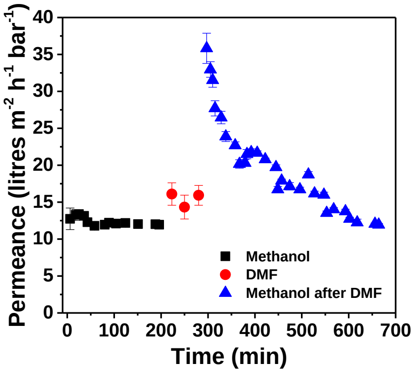


**Supplementary Figure 45 | Impact of DMF post treatment.** Methanol permeance before and after dimethylformamide (DMF) treatment for the composite membrane comprising nanofilms ($\beta$-CDA-TPC-0.1-Al) made from 0.1 wt.% $\beta$-CDA and 0.1 wt.% TPC and then transferred onto alumina supports. No activation by DMF was observed for the membrane.

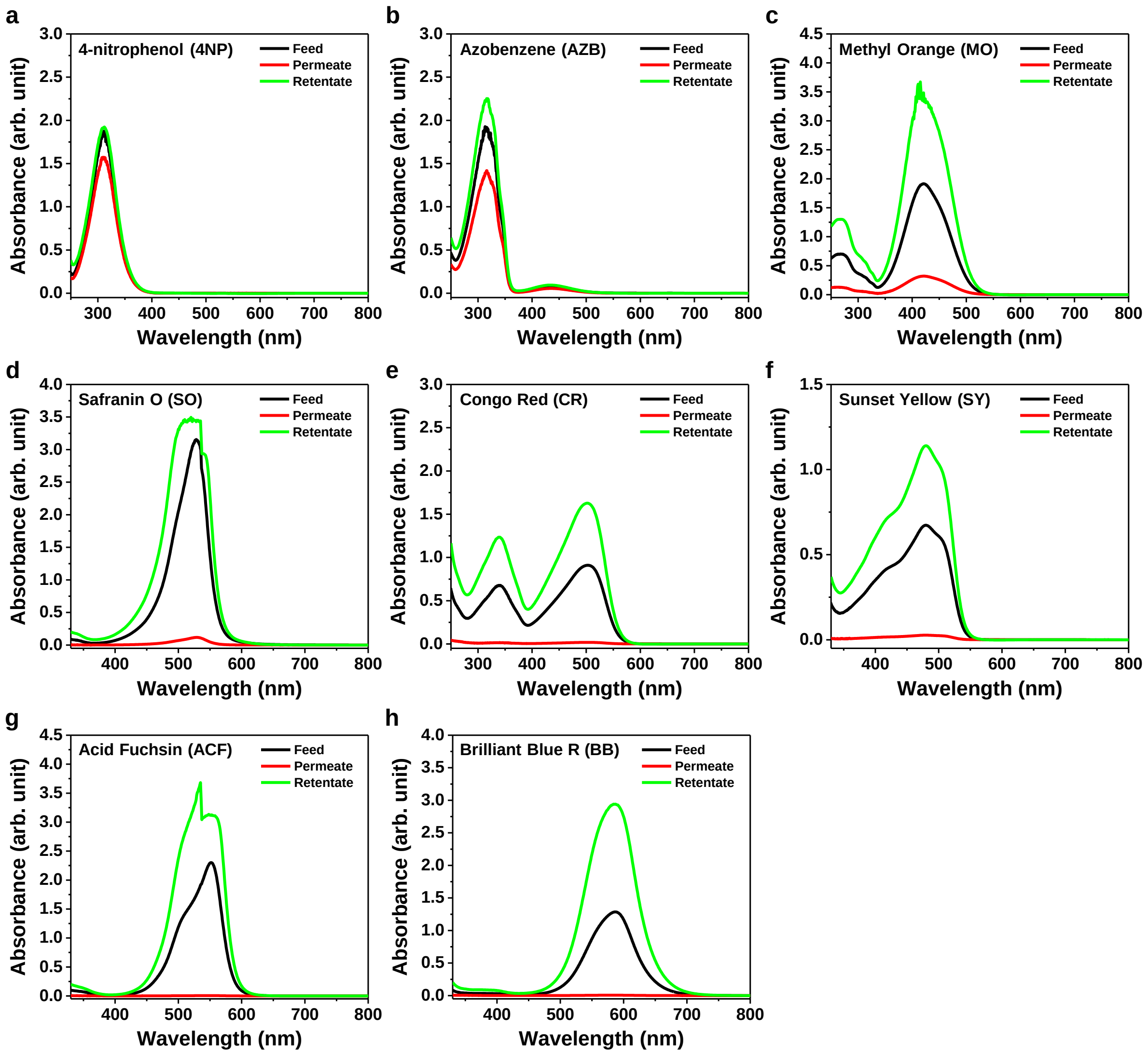


**Supplementary Figure 46 | Typical ultraviolet-visible (UV-vis) absorption spectra of dyes in feed, permeate, and retentate demonstrating the rejection performance of composite membranes.** This membrane comprised nanofilms (*β*-CDA-TPC-0.1-Al) made from 0.1 wt.% *β*-CDA and 0.1 wt.% terephthaloyl chloride (TPC) at the free interface for 1 min and then transferred onto alumina supports.

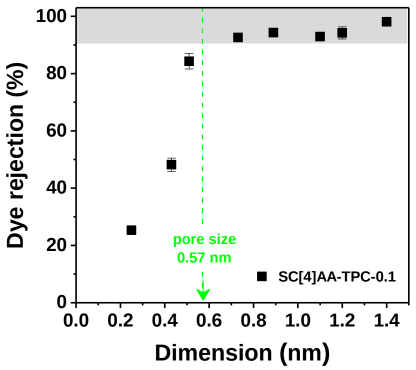


**Supplementary Figure 47 | Dye rejections of the composite membranes.** They comprise nanofilms made from amino-functionalised 4-sulfocalix[4]arene sodium salt (SC[4]AA) and 0.1 wt.% terephthaloyl chloride (TPC) at the free interface for 20 min and then transferred onto PAN supports.

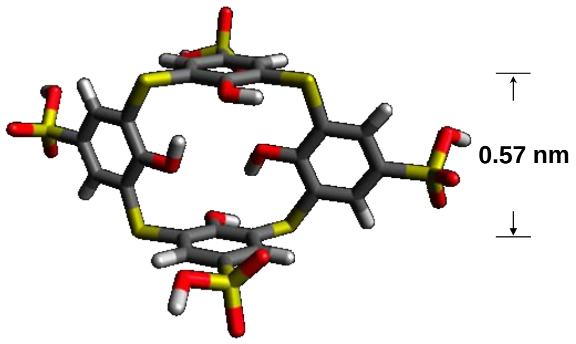


**Supplementary Figure 48 | Schematic illustrating the molecular dimensions of amino-functionalised 4-sulfocalix[4]arene sodium salt (SC[4]AA).**

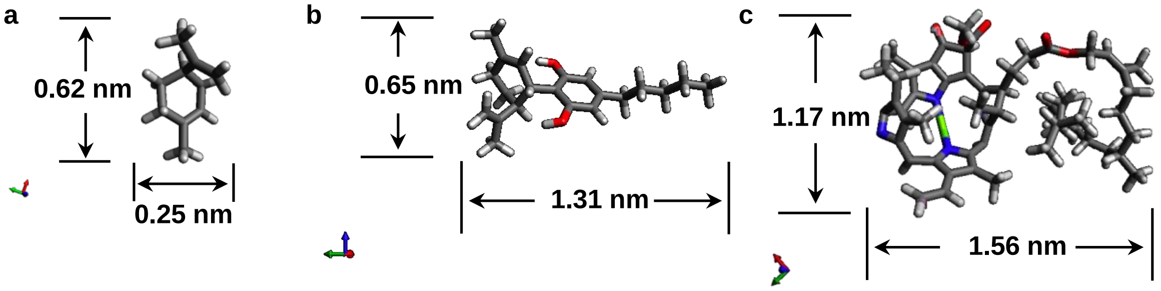


**Supplementary Figure 49 | Schematic illustrating the molecular dimensions. a,** limonene (136 g mol$^{-1}$), **b,** CBD (314 g mol$^{-1}$), and **c,** chlorophyll (893 g mol$^{-1}$).

**Supplementary Table 6.** Comparison of selectivity performance between membranes reported in literature and membranes fabricated from amino-functionalised macrocycles in this work.

| Membrane material | Methanol permeance ($L\ m^{-2}\ h^{-1}\ bar^{-1}$) | MW <300 $g\ mol^{-1}$ | | MW 300-400 $g\ mol^{-1}$ | | MW 400-500 $g\ mol^{-1}$ | | Selectivity | | Ref. |
|---|---|---|---|---|---|---|---|---|---|---|
| | | Dye (MW) | Rejection (%) | Dye (MW) | Rejection (%) | Dye (MW) | Rejection (%) | MW<300 / MW 300-400 | MW 300-400 / MW 400-500 | |
| Cyclodextrin | 4.9 | | | MO (327) | 67 | IC (466) | 88 | - | 2.75 | 8 |
| Trianglamine macrocycle | 22 | | | MO (327) | 83 | OG (452) | 96 | - | 4.25 | 9 |
| COF | 72 | NR (229) | 2 | SO (351) | 5 | PR (453) | 5 | 1.03 | 1.00 | 13 |
| Cyclodextrin | 17 | MR (291) | 60 | MO (327) | 88 | RBB (442) | 95 | 1.58 | 2.40 | 17 |
| Polyarylate | 8 | CSG (249) | 70 | DR (314) | 90 | CV (408) | 97 | 3.00 | 3.33 | 30 |
| Graphene oxide | 9 | HNSA (246) | 99.9 | DR (314) | 99.9 | CV (408) | 99.9 | 1.00 | 1.00 | 31 |
| Polyimide | 1.6 | | | MO (327) | 60 | OG (452) | 82 | - | 2.22 | 32 |
| Cyclodextrin | 5.8 | MR (291) | 60 | MO (327) | 93 | | | 5.71 | - | 7 |
| PIM | 8.7 | SOG (214) | 99.9 | DR (314) | 99.9 | | | 1.00 | - | 33 |
| Conjugated polymers | 22.5 | AZB (182) | 10.2 | MB (320) | 48.4 | | | 1.74 | - | 34 |
| $\gamma$-CDA-TPC-0.1 | 9.30 | | | MO (327) | 60.0 | SY (452) | 94.5 | - | 7.27 | This work |
| $\beta$-CDA-TPC-0.05 | 9.84 | | | MO (327) | 81.3 | SY (452) | 98.3 | - | 11 | This work |
| $\alpha$-CDA-TPC-0.05 | 7.64 | AZB (182) | 33.9 | MO (327) | 93.0 | | | - | 9.44 | This work |
| $\alpha$-CDA-TPC-0.1-Al | 9.51 | AZB (182) | 18.3 | MO (327) | 91.3 | | | - | 9.39 | This work |

Abbreviations: MW-molecular weight, COF-covalent organic frameworks, PIM-polymers of intrinsic microporosity, AZB-azobenzene, SOG-sudan orange G, NR-natural red, HNSA-6-hydroxy-2-naphthalenesulfonic acid sodium salt, CSG-chrysoidine G, MR-methyl red sodium salt, DR-disperse red, MB-methylene blue, MO-methyl orange, SO-safranin O, CV-crystal violet, RBB-rhodamine B base, SY-sunset yellow, OG-orange G, PR-primuline, IC-indigo carmine. The molecular weight of each dye was shown in the bracket after the name of the dyes. Selectivity was calculated by following Equation 2.

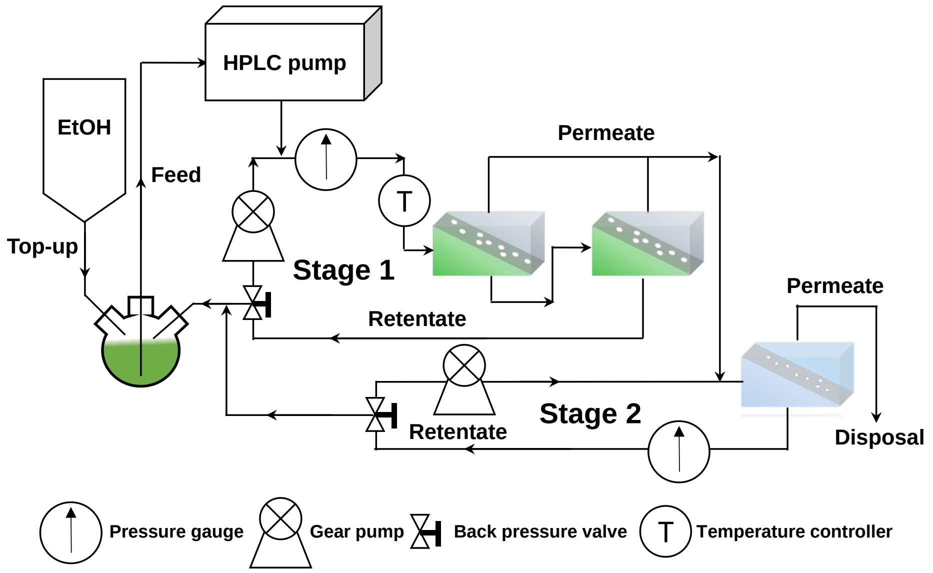


**Supplementary Figure 50 | Schematic illustrating a cascade process for enriching CBD.**

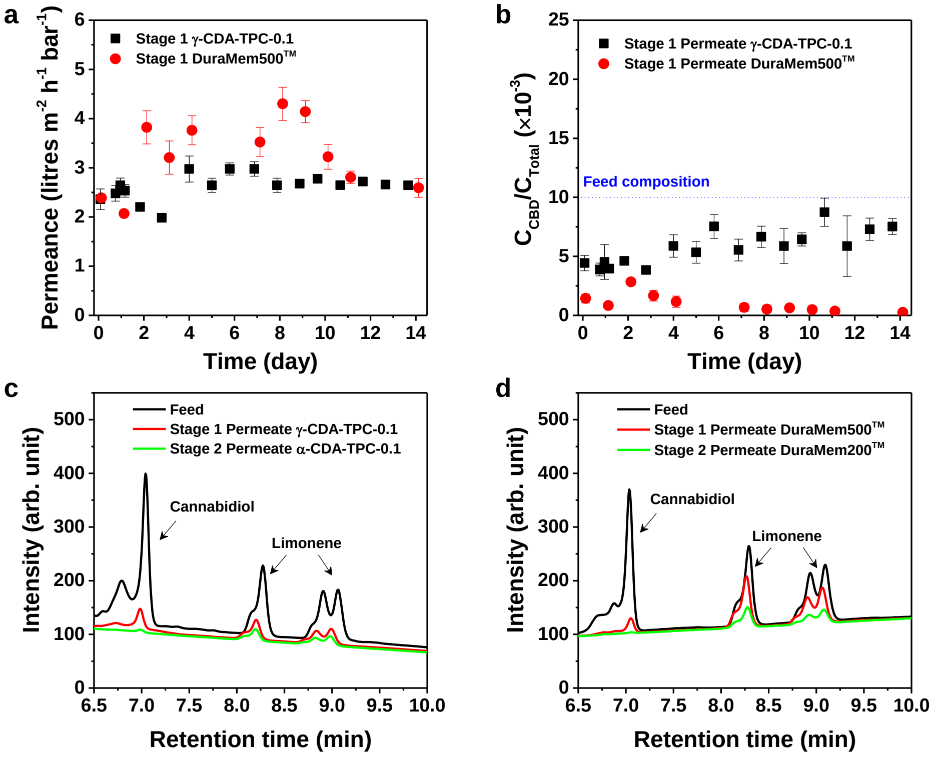


**Supplementary Figure 51 | Performance of cascade process enriching CBD. a,** Ethanol permeance and **b,** the concentration of CBD in permeate over time for nanofilms incorporating aligned macrocycle pores (*γ*-CDA-TPC-0.1) and commercial standard membrane DuraMem500™ used in Stage 1. Ultra-violet (UV) absorbance of CBD and limonene from high-performance liquid chromatography (HPLC) for **c,** composite membranes incorporating aligned macrocycle pores versus **d,** commercial membranes. Composite membranes comprising large cavity (*γ*-CDA-TPC-0.1) and DuraMem500™ were used at Stage 1 and composite membranes comprising small cavity (*α*-CDA-TPC-0.1) and DuraMem200™ were used at Stage 2.

## References


41. Zhao, Q. & Liu, Y., Macrocycle crosslinked mesoporous polymers for ultrafast separation of organic dyes. *Chem. Commun.* **54**, 7362-7365 (2018).

42. van de Manakker, F., Vermonden, T., van Nostrum, C. F. & Hennink, W. E., Cyclodextrin-Based Polymeric Materials: Synthesis, Properties, and Pharmaceutical/Biomedical Applications. *Biomacromolecules* **10**, 3157-3175 (2009).

43. Hansen, C. M., *Hansen Solubility Parameters: A User's Handbook* (CRC Press, Boca Raton, FL, ed. 2, 2007).
